# Obstructive Sleep Apnea and Risk of Cardiovascular Events and All-Cause Mortality: A Decade-Long Historical Cohort Study

**DOI:** 10.1371/journal.pmed.1001599

**Published:** 2014-02-04

**Authors:** Tetyana Kendzerska, Andrea S. Gershon, Gillian Hawker, Richard S. Leung, George Tomlinson

**Affiliations:** 1 Institute of Health Policy, Management and Evaluation, Faculty of Medicine, University of Toronto, Toronto, Ontario, Canada; 2 Department of Medicine, Faculty of Medicine, University of Toronto, Toronto, Ontario, Canada; 3 Institute for Clinical Evaluative Sciences, Toronto, Ontario, Canada; 4 Department of Medicine, Sunnybrook Health Sciences Centre, Toronto, Ontario, Canada; 5 Department of Medicine, Women's College Hospital, Toronto, Ontario, Canada; 6 Sleep Laboratory, St. Michael's Hospital, Toronto, Ontario, Canada; 7 Department of Medicine, St. Michael's Hospital, Toronto, Ontario, Canada; 8 Department of Medicine, University Health Network/Mt Sinai Hospital, Toronto, Ontario, Canada; The George Institute for Global Health, Australia

## Abstract

**Background:**

Obstructive sleep apnea (OSA) has been reported to be a risk factor for cardiovascular (CV) disease. Although the apnea-hypopnea index (AHI) is the most commonly used measure of OSA, other less well studied OSA-related variables may be more pathophysiologically relevant and offer better prediction. The objective of this study was to evaluate the relationship between OSA-related variables and risk of CV events.

**Methods and Findings:**

A historical cohort study was conducted using clinical database and health administrative data. Adults referred for suspected OSA who underwent diagnostic polysomnography at the sleep laboratory at St Michael's Hospital (Toronto, Canada) between 1994 and 2010 were followed through provincial health administrative data (Ontario, Canada) until May 2011 to examine the occurrence of a composite outcome (myocardial infarction, stroke, congestive heart failure, revascularization procedures, or death from any cause). Cox regression models were used to investigate the association between baseline OSA-related variables and composite outcome controlling for traditional risk factors. The results were expressed as hazard ratios (HRs) and 95% CIs; for continuous variables, HRs compare the 75th and 25th percentiles. Over a median follow-up of 68 months, 1,172 (11.5%) of 10,149 participants experienced our composite outcome. In a fully adjusted model, other than AHI OSA-related variables were significant independent predictors: time spent with oxygen saturation <90% (9 minutes versus 0; HR = 1.50, 95% CI 1.25–1.79), sleep time (4.9 versus 6.4 hours; HR = 1.20, 95% CI 1.12–1.27), awakenings (35 versus 18; HR = 1.06, 95% CI 1.02–1.10), periodic leg movements (13 versus 0/hour; HR = 1.05, 95% CI 1.03–1.07), heart rate (70 versus 56 beats per minute [bpm]; HR = 1.28, 95% CI 1.19–1.37), and daytime sleepiness (HR = 1.13, 95% CI 1.01–1.28).The main study limitation was lack of information about continuous positive airway pressure (CPAP) adherence.

**Conclusion:**

OSA-related factors other than AHI were shown as important predictors of composite CV outcome and should be considered in future studies and clinical practice.

*Please see later in the article for the Editors' Summary*

## Abstract 2

Tetyana Kendzerska and colleagues explore the association between physiological measures of obstructive sleep apnea other than the apnea-hypopnea index and the risk of cardiovascular events.

*Please see later in the article for the Editors' Summary*

## Editors' Summary

**Background:**

Obstructive sleep apnea (OSA) is a common sleep-related breathing disorder, particularly among middle-aged and elderly people. It is characterized by apnea—a brief interruption in breathing that lasts at least 10 seconds—and hypopnea—a decrease of more than 50% in the amplitude of breathing that lasts at least 10 seconds or clear but smaller decrease in amplitude associated with either oxygen desaturation or an arousal. Patients with OSA experience numerous episodes of apnea and hypopnea during the night; severe OSA is defined as having 30 or more episodes per hour (an apnea-hypopnea index [AHI] of >30). These breathing interruptions occur when relaxation of the upper airway muscles decreases the airflow, which lowers the amount of oxygen in the blood. As a result, affected individuals frequently wake from deep sleep as they struggle to breathe. Symptoms of OSA include loud snoring and daytime sleepiness. Treatments include lifestyle changes such as losing weight (excess fat around the neck increases airway collapse) and smoking cessation. For severe OSA, doctors recommend continuous positive airway pressure (CPAP), in which a machine blows pressurized air through a face mask into the airway to keep it open.

**Why Was This Study Done?:**

OSA can be life-threatening. Most directly, daytime sleepiness can cause accidents, but OSA is also associated with an increased risk of developing cardiovascular disease (CVD, disease that affects the heart and the circulation). To date, studies that have investigated the association between OSA and the risk of myocardial infarction (heart attack), congestive heart failure, stroke, and other CVDs have used the AHI to diagnose and categorize the severity of OSA. However, by focussing on AHI, clinicians and researchers may be missing opportunities to improve their ability to predict which patients are at the highest risk of CVD. In this historical cohort study, the researchers investigate the association between other OSA-related variables (for example, blood oxygen saturation and sleep fragmentation) and the risk of cardiovascular events and all-cause mortality (death). A historical cohort study examines the medical records of groups of individuals who have different characteristics at baseline for the subsequent occurrence of specific outcomes.

**What Did the Researchers Do and Find?:**

The researchers used administrative data (including hospitalization records and physicians' claims for services supplied to patients) to follow up adults referred for suspected OSA who underwent diagnostic polysomnography (a sleep study) at a single Canadian hospital between 1994 and 2010. A database of the polysomnography results provided information on OSA-related variables for all the study participants. Over an average follow-up of about 6 years, 11.5% of the 10,149 participants were hospitalized for a myocardial infarction, stroke, or congestive heart failure, underwent a revascularization procedure (an intervention that restores the blood supply to an organ or tissue after CVD has blocked a blood vessel), or had died from any cause. After adjusting for multiple established risk factors for CVD such as smoking and age in Cox regression models (a statistical approach that examines associations between patient variables and outcomes), several OSA-related variables (but not AHI) were significant predictors of CVD. The strongest OSA-related predictor of cardiovascular events or all-cause mortality was total sleep time spent with oxygen saturation below 90%, which increased the risk of a cardiovascular event or death by 50%. Other statistically significant OSA-related predictors (predictors that were unlikely to be associated with the outcome through chance) of cardiovascular events or death included total sleep time, number of awakenings, frequency of periodic leg movements, heart rate, and daytime sleepiness.

**What Do These Findings Mean?:**

These findings indicate that OSA-related factors other than AHI are important predictors of the composite outcome of a cardiovascular event or all-cause mortality. Indeed, although AHI was significantly associated with the researchers' composite outcome in an analysis that did not consider other established risk factors for CVD (“confounders”), the association became non-significant after controlling for potential confounders. The accuracy of these findings, which need to be confirmed in other settings, is likely to be limited by the lack of information available about the use of CPAP by study participants and by the lack of adjustment for some important confounders. Importantly, however, these findings suggest that OSA-related factors other than AHI should be considered as predictors of CVD in future studies and in clinical practice.

**Additional Information:**

Please access these websites via the online version of this summary at http://dx.doi.org/10.1371/journal.pmed.1001599.

- The US National Heart Lung and Blood Institute has information (including several videos) about obstructive sleep apnea (in English and Spanish), sleep studies, heart disease, and other cardiovascular diseases (some information in English and Spanish)
- The UK National Health Service Choices website provides information (including personal stories) about sleep apnea and about cardiovascular disease
- The not-for-profit American Sleep Apnea Association provides detailed information about sleep apnea for patients and health-care professionals, including personal stories about the condition
- The MedlinePlus encyclopedia has pages on obstructive sleep apnea and on polysomnography; MedlinePlus provides links to further information and advice about obstructive sleep apnea, heart diseases, and vascular diseases (in English and Spanish)

## Introduction

Obstructive sleep apnea (OSA) is a sleep-related breathing disorder that is characterized by repeated episodes of upper airway occlusion during sleep. OSA afflicts 3%–9% of women and 10%–17% of men in the United States [1]. Sleep apnea can increase the risk of developing cardiovascular (CV) disease through a number of mechanisms, including intermittent hypoxia, sleep fragmentation, chronic sympathetic activation, and systemic inflammation [2]–[6].

Although evidence exists for relationships between OSA and both all-cause mortality and various CV events, uncertainty surrounds the magnitude of these associations, and the contributions of different OSA-related variables to the development of long-term adverse outcomes [7]. The apnea-hypopnea index (AHI) is the most often-reported statistically significant predictor; however, a variety of non-clinically determined AHI thresholds are arbitrarily used to diagnose and categorize severity of OSA [7],[8]. By focusing almost exclusively on AHI, clinicians and researchers may have missed opportunities to better risk-stratify patients using other OSA-related variables [9]. A number of less-studied variables may be more pathophysiologically relevant and offer better predictive ability than AHI, which is only a crude measure of breathing stoppages: intermittent hypoxemia (e.g., level of oxygen saturation [SaO_2_]), sleep fragmentation or sleep deprivation (e.g., total sleep time [TST] or number of awakenings), sympathetic activation (e.g., heart rate during sleep), symptoms (daytime sleepiness, snoring), family history of snoring or OSA, and findings from physical examination (neck circumference) [7],[10].

A number of large, well-designed community-based studies have examined the relationship between OSA and CV disease and mortality [11]–[14]. However, these have been limited by the small number of individuals with severe OSA. On the other hand, in clinically based studies with higher disease severity, small numbers of events limit the number of variables that can be included in statistical models, a weak definition of CV events is often used, women are usually underrepresented, and inconsistency in polysomnographic scoring criteria over time means long-term follow-up is not possible [7].

Our study addresses the weaknesses described above by following up a large number of individuals with a wide spectrum of severity of OSA and evaluating the relationship between a comprehensive set of OSA-related variables and the development of CV outcomes and all-cause mortality after controlling for traditional CV risk factors. Finally, our study aims to resolve conflicting evidence on the impact of gender, age, body mass index (BMI), daytime sleepiness (DS), and comorbid CV disease on the strength of association between OSA and development of CV outcomes.

We hypothesize that the AHI, currently used to establish severity of OSA, is not by itself sufficient to accurately predict CV outcomes in individuals with OSA. We also hypothesize that an expanded set of factors including patient demographic and clinical characteristics and physiologic indices will provide greater accuracy in predicting CV outcomes.

## Methods

### Study Design

A historical cohort study was conducted using a clinical sleep database and provincial health administrative data. All adults who underwent a first diagnostic sleep study at St. Michael's Hospital (Toronto, Ontario, Canada) between September 1, 1994 and December 31, 2010 and were diagnosed or referred with OSA were included. Their clinical data were linked to health administrative data at the Institute for Clinical Evaluative Sciences (ICES, Toronto, Ontario, Canada) from July 1, 1991 to March 31, 2011.

### Ethics Statement

The ethics committees of all institutions involved (St. Michael's Hospital, ICES, University of Toronto) approved the study.

### Data Sources

#### Clinical data

The St. Michael's Hospital database includes a large set of clinical, demographic, and polysomnographical (PSG) variables that have been collected consistently for research purposes since 1991 (Table S1). Each patient in the cohort underwent full in-laboratory PSG recording that was scored by a sleep technologist and reviewed by a sleep physician. Disease-specific symptoms and history were collected using standardized questionnaires; a physical examination was performed by sleep technicians.

#### Health administrative data

Residents of Ontario have universal public health insurance covering all medically necessary services. ICES houses high quality [15] administrative data on a wide variety of publicly funded services provided since 1991, including individual-level information on physician claims, acute care hospitalization, and emergency department visits within the province. For Ontario residents with diagnosed OSA, funding is provided for continuous positive airway pressure (CPAP) devices, and this funding is documented in the Assistive Devices Program (ADP) database from 2004 onwards [16]. Table S2 gives details of variables derived from administrative data sets.

### Study Sample

Patients who had undergone a first diagnostic sleep study during the defined study period, and who had a diagnosis of OSA (AHI≥5), or suspected OSA (referred with sleep apnea, but with AHI<5) were extracted from the St. Michael's Hospital database. Patients were excluded if they had (i) more than 50% central events or (ii) AHI<5 and a diagnosis of another sleep disorder.

### Predictors

The following OSA-related variables were derived from clinical data and considered as predictors in our statistical models: (i) PSG indexes—TST, the percentage of each sleep stage, AHI, apnea index, hypopnea index, mean duration of apnea and hypopnea, total arousals index, total number of awakenings; mean of SaO_2_ in TST, sleep time spent with SaO_2_<90% (TST90SaO_2_); periodic leg movements index; and mean heart rate; (ii) clinical symptoms—DS, identified by means of the Epworth Sleepiness Scale or a positive answer to the question “During the day, do you ever fall asleep unintentionally?”; self-reported snoring; and morning headache; (iii) neck circumference; (iv) self-reported family history of snoring or OSA.

The AHI was defined as the number of apneas and hypopneas per hour of sleep. The definition of hypopnea was consistent during the study period: (i) a decrease of more than 50% of the baseline amplitude of breathing lasting 10 seconds or longer; or (ii) a clear but smaller decrease in amplitude lasting for at least 10 seconds that is associated with either an SaO_2_ drop of ≥3% or an arousal [17]. OSA was classified as mild (AHI of 5 to 14.9), moderate (AHI of 15 to 30), or severe (AHI>30) [18].

We assumed that CPAP treatment started at the time of the claim in the ADP data set.

### Outcome Variables

The primary composite outcome was defined using health administrative data as the first of (i) hospitalization due to myocardial infarction (MI), stroke, or exacerbation of congestive heart failure (CHF); (ii) a revascularization procedure (percutaneous coronary intervention, coronary artery bypass graft surgery); or (iii) all-cause death (Table S2). Hospitalization was chosen as a well-defined, validated, and standardized measure of interest to patients, doctors, and policy-makers. Participants were followed from their first diagnostic sleep study to the end of March 2011, or the occurrence of a primary outcome, whichever occurred first.

### Potential Confounders and Risk Factors

The following potential confounders and risk factors were extracted from clinical data: age, sex, BMI, waist and hip circumferences, and self-reported smoking. Comorbidities at baseline (stroke, MI, CHF, hypertension [HTN], chronic obstructive pulmonary disease [COPD], depression, and diabetes) were identified from administrative data over a three-year period before the diagnostic sleep study. Neighbourhood income and rural status were derived from administrative data at the time of the diagnostic sleep study.

### Statistical Analysis

Descriptive statistics were calculated for relevant data. Crude incidence rates of the composite end point per 100 person-years were calculated for each OSA severity group.

Since AHI is the standard for defining of OSA and its severity [2],[18], and to allow comparisons with other research in this area, event-free survival in OSA severity groups was estimated using the Kaplan–Meier method and compared between groups with the log-rank test.

We used univariate and multivariable Cox regression models to investigate the relationships between additional predictors and the CV outcome, and expressed the results as hazard ratios (HRs) and 95% CIs. To avoid choosing arbitrary cut points for PSG characteristics (e.g., AHI, TST), they were kept as continuous variables. We used restricted cubic spline transformations for continuous explanatory variables if non-linearity was observed (Figure S1), and the resulting standardized HRs compare the 75th and 25th percentiles, allowing comparison of the HRs on a common scale. The proportional hazards assumption for each variable was tested [19],[20].

Variables missing on more than 50% of individuals were excluded from all analyses (Table S1). For all others, we used multivariate imputation by chained equations to generate five complete data sets [21]. Eighty two variables including both the event status and the survival time were chosen for the imputation model. The following built-in imputation models were used in our analyses: for continuous variables, predictive mean matching; for binary variables, logistic regression; for unordered categorical variables, polytomous logistic regression; and for ordered categorical variables, proportional odds [22]. The separate estimates and standard errors from imputed data sets were pooled according to Rubin's rules [23]. For a unified presentation of all results and figures, the findings shown are for a single imputed data set. Pooled CIs across imputations were at most 2% wider than those presented.

A systematic review [7] and expert opinion found age, sex, smoking status, BMI, AHI, TST, and DS to be clinically important, so these variables were forced into the models. Other variables were chosen for inclusion in the final model if they were selected by backward step-down variable deletion [24] in at least three imputed data sets. We investigated *a priori*-defined interactions between AHI or SaO_2_ and DS, BMI, age, sex, and CVD at baseline [7]. The final model was compared to a model with only traditional CV risk factors (age, sex, smoking status, BMI, prior HTN, diabetes, MI, stroke, and CHF [25]) using a likelihood ratio test [19]. To identify which outcomes were driving results, the final model was refitted to each separate component of the composite CV outcome.

A clinical nomogram was constructed from the multivariable Cox model to estimate three- and five-year event-free survival probabilities and median survival times [26].

We used the bootstrap for internal validation (optimism for C-index and R^2^) and over-fitting-corrected calibration (predicted versus observed five-year survival). Discriminative ability was assessed using Harrell's C-index and predictive ability using the model likelihood ratio χ^2^ statistic [19].

#### Sensitivity analysis

In the post-2004 cohort with information on CPAP claims, the final model was refitted with the addition of a time-dependent CPAP treatment variable.

All statistical analyses were performed using R version 2.15.2 (http://www.r-project.org) and SAS 9.2.

## Results

### Sample Characteristics

**Figure 1:**
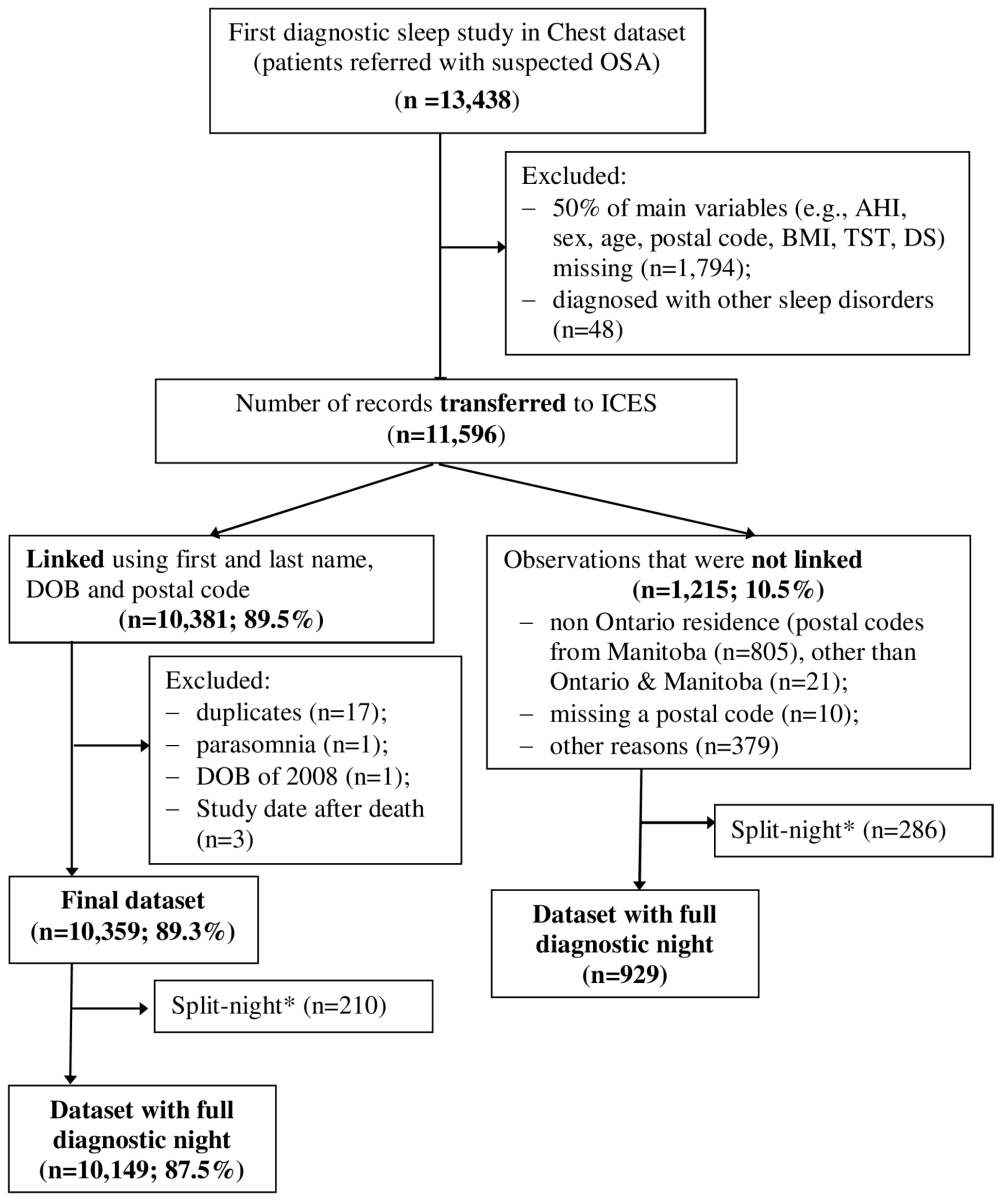
Flow diagram of the final cohort. *Split night, diagnostic study night when treatment was initiated due to severe OSA.

Between January 1, 1994 and December 31, 2010, 11,596 individuals underwent a first diagnostic sleep study and 10,149 (88%) were linked to administrative data sets and included in our analyses (Figure 1). Table 1 shows baseline characteristics of included and excluded patients. Excluded patients had similar OSA severity and demographic characteristics, but fewer CV comorbidities and greater DS. The included sample had 62% males, a mean age of 50 years, and a mean AHI of 25. The amount of missing data ranged from 0.69% (AHI) to10.1% (TST90SaO_2_).

**Table 1:** Comparison of included and excluded samples for patients with a full-night diagnostic sleep study.

Variables	Included (*n* = 10,149)		Excluded (*n* = 929)	
	Mean (SD) Unless Otherwise Indicated	Missing, *n* (%)	Mean (SD) Unless Otherwise Indicated	Missing, *n* (%)
***Demographic characteristics***				
Male	6,288 (62.0)	—	544 (58.6)	—
Age, years	49. 9 (14.1)	—	46.7 (13.2)	14 (1.5)
***Clinical symptoms and findings from physical examination***				
“During the day, do you ever fall asleep unintentionally?” Yes, *n* (%)	3,657 (36.0)	688 (6.8)	382 (41.1)	54 (5.8)
ESS total (0–24)^a^	8.6 (5.0)	615 (6.0)	10.3 (5.3)	40 (4.3)
BMI, kg/m^2^	30.1 (7.0)	243 (2.4)	30.7 (6.9)	12 (1.3)
***History***				
Smoking status, self-reported, *n* (%)		835 (8.2)		68 (7.3)
Current	1,839 (18.1)	—	174 (18.7)	—
Ex-smoker	1,869 (18.4)	—	164 (17.7)	—
Never	5,606 (55.2)	—	523 (56.3)	—
MI, self-reported, Yes, *n* (%)^a^	605 (6.0)	640 (6.3)	22 (2.4)	44 (4.7)
CABG, self-reported, Yes, *n* (%)^a^	322 (3.2)	544 (5.4)	11 (1.2)	38 (4.1)
Stroke, self-reported, Yes, *n* (%)^a^	301 (3.0)	546 (5.4)	16 (1.7)	38 (4.1)
HTN, self-reported, Yes, *n* (%)^a^	3,097 (30.5)	585 (5.8)	250 (26.9)	44 (4.7)
Lung disease, self-reported, Yes, *n* (%)	1,697 (16.7)	609 (6.0)	157 (16.9)	46 (5.0)
***PSG indexes***				
TST, hours	5.5 (1.3)	241 (2.4)	5.9 (1.2)	14 (1.5)
Sleep efficiency, %	76.5 (18.1)	211 (2.1)	78.6 (16.0)	23 (1.9)
AHIO in TST, events/hour	21.1 (23.1)	70 (0.7)	21.6 (24.5)	7 (0.8)
AHI in TST, events/hour	24.6 (25.3)	70 (0.7)	24.0 (26.0)	7 (0.8)
OSA, severity, *n* (%)		70 (0.7)		7 (0.8)
No OSA: AHI<5	2,109 (20.8)	—	225 (24.2)	—
Mild: 5≤AHI<15	2,703 (26.6)	—	252 (27.1)	—
Moderate:15≤AHI≤30	2,307 (22.7)	—	187 (20.1)	—
Severe: >30	2,960 (29.2)	—	258 (27.8)	—
Arousals index, total, events/hour	29.9 (23.0)	511 (5.0)	30.7 (24.3)	34 (3.7)
AWK in TST, number of events	28.8 (18.5)	214 (2.1)	28.7 (17.2)	11 (1.2)
TST90SaO_2_, minutes	18.9 (49.7)	1,022 (10.1)	19.5 (51.9)	25 (2.7)
Mean SaO_2_, %	94.4 (3.4)	60 (0.6)	94.4 (4.0)	5 (0.5)
Heart rate, mean/TST, bpm	63.7 (10.3)	794 (7.8)	64.5 (10.7)	61 (6.6)

a The differences between groups considered as clinically important as indicated by a systematic review [7] and expert opinion.

AHIO, total and obstructive apnea-hypopnea index; ArI, total arousals index; AWK, total number of awakenings; bpm, beats per minute; CABG, coronary artery bypass graft surgery; ESS, Epworth Sleepiness Scale; TST90SaO_2_, sleep time spent with SaO_2_ less than 90%.

Over a median follow-up of 68 months, 1,172 (11.5%) participants experienced a composite outcome, giving an incidence rate of 2 per 100 person-years. Event-free survival was 94% at three years and 90% at five years with CIs less than ±0.5%.

### Analyses of AHI

**Figure 2:**
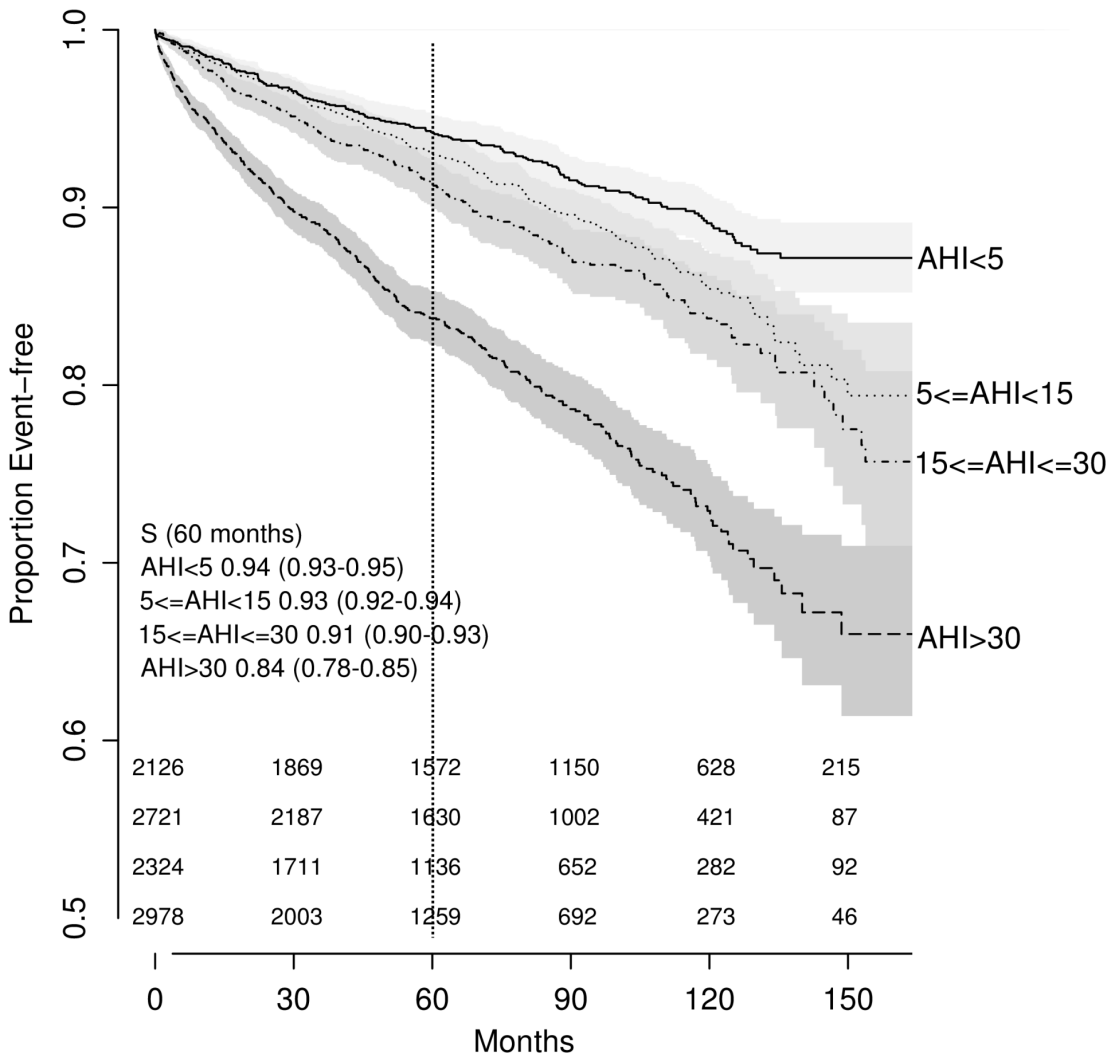
Unadjusted Kaplan-Meier survival curves by obstructive sleep apnea severity as expressed by the apnea-hypopnea index. The numbers at risk are presented above the x-axis: from the top to the bottom, AHI<5; 5≤AHI<15; 15≤AHI<30; AHI>30.

**Figure 3:**
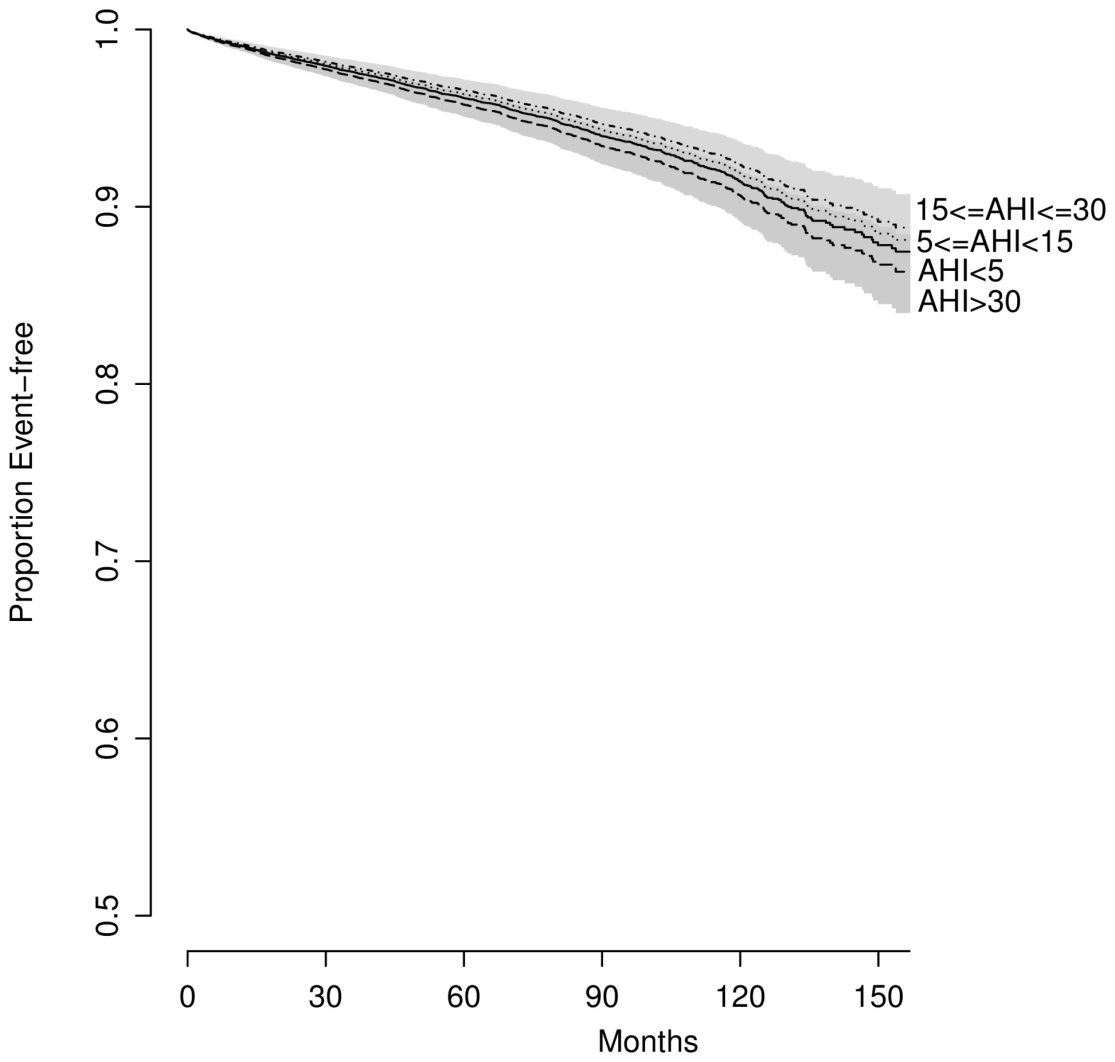
Predicted survival by OSA severity, adjusted for traditional CV risk factors (BMI = 29, age = 50, sex = men, never smoked, without prior hypertension, diabetes, MI, stroke, or heart failure).

AHI was significantly associated with event-free survival in univariate analyses when categorized (log-rank test; *p*<0.001; Figure 2) or treated as a continuous predictor in a Cox model (35 versus 6.3 events/hour: HR = 1.49, 95% CI 1.42–1.57; *p*<0.001). After controlling for traditional CV risk factors, the magnitude of association was attenuated: no significant difference was found across OSA severity groups (*p*>0.2) (Figure 3), although the association between AHI as a continuous variable and outcome of interest remained significant (HR = 1.12; 95% CI 1.05–1.2, *p*<0.001).

### Multivariable Cox Regression Models

**Figure 4:**
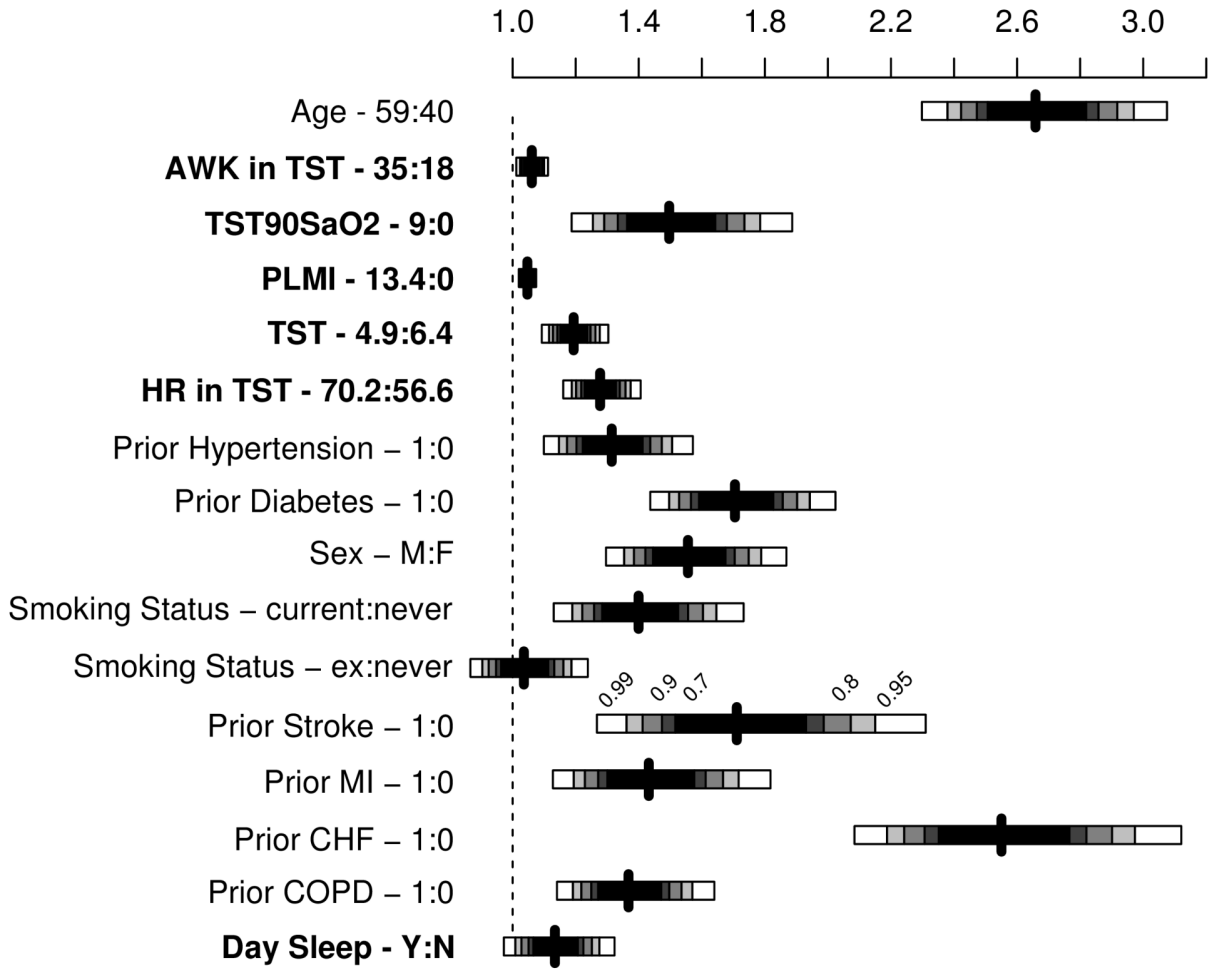
Results from multivariable Cox regression model presented as hazard ratios with shading representing confidence levels (99%, 95%, 90%, 80%, and 70%). AWK, number of awakenings in TST; TST90SaO_2_, sleep time spent with SaO_2_ less than 90%; PLMI, periodic leg movement index; HR, mean heart rate during sleep; day sleep, DS, identified by a positive answer to the question “During the day, do you ever fall asleep unintentionally?”.

**Figure 5:**
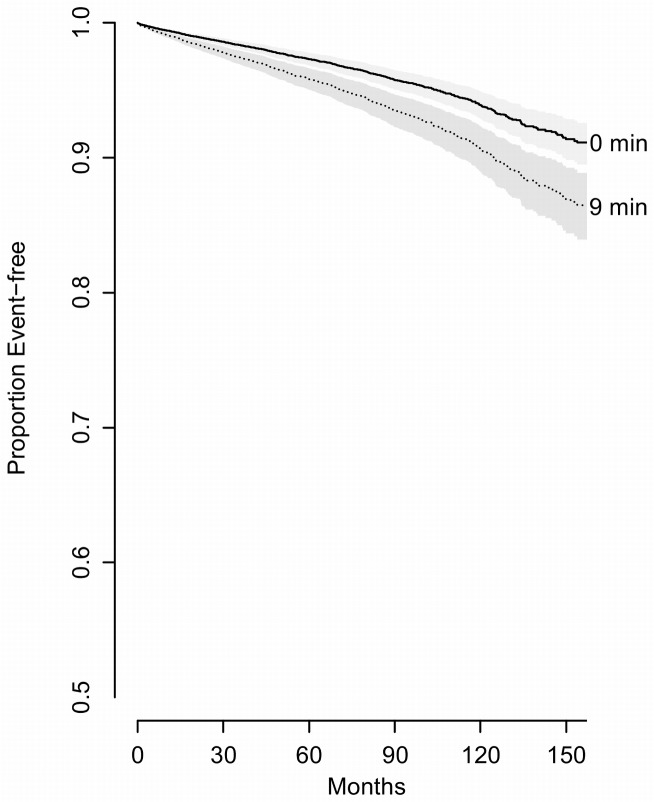
Predicted survival curves to show the effect of oxygen saturation (comparing 75th percentile [9 min] to 25th percentile [0 min]) controlling for potential confounders (BMI = 29, age = 50, sex = men, never smoked, without prior hypertension, diabetes, MI, stroke or heart failure, TST = 5.8, AWK = 25, PLMI, 1.2, mean heart rate, 63, without reporting excessive DS).

In the fully adjusted model, the following OSA-related predictors were significantly associated with occurrence of a composite outcome: TST90SaO_2_, TST, awakenings, periodic leg movements, mean heart rate, and presence of unintentional DS (Figures 4 and 5; Table 2). AHI was no longer a significant predictor.

**Table 2:** Model fitting and effect of predictors (*n* = 10,149, events = 1,172).

Variable (X:Y) Indicates the Two Values Being Compared	Univariate Models		Multivariable Models			
	HR (95% CI)	LR χ^2^ (R^2^)	CV Risk Factors	Final Model (without Transformation)	Final Model (with Transformation^a^)	
***Demographic characteristics***						
Sex (M:F)	1.71 (1.5–1.94)	69.6 (0.008)	1.62 (1.41–1.85)	1.60 (1.39–1.82)	1.55 (1.35–1.79)	
Age, years (59:40)	4.54 (4.18–4.94)	1,337.3 (0.143)	3.27 (2.95–3.62)	2.70 (2.42–3.01)	2.66 (2.38–2.97)	
BMI (33.6:25.4)	1.25 (1.18–1.33)	48.9 (0.006)	1.17 (1.09–1.25)	—	—	
***History***						
Smoking Status		109.3 (0.012)				
ex-smoker:never	2.03 (1.79–2.32)	—	1.13 (0.99–1.30)	1.04 (0.91–1.20)	1.04 (0.90–1.19)	
current:never	1.13 (0.97–1.32)	—	1.70 (1.45–1.99)	1.41 (1.20–1.66)	1.40 (1.19–1.65)	
Prior comorbidities						
HTN (Yes:No)	4.12 (3.65–4.65)	564.3 (0.062)	1.40 (1.22–1.61)	1.36 (1.18–1.55)	1.31 (1.15–1.51)	
Diabetes (Yes:No)	4.53 (4.01–5.11)	492.4 (0.055)	1.89 (1.66–2.16)	1.71 (1.50–1.95)	1.71 (1.50–1.94)	
Stroke (Yes:No)	4.96 (3.97–6.20)	130.7 (0.015)	1.61 (1.28–2.02)	1.71 (1.36–2.15)	1.71 (1.36–2.15)	
MI (Yes:No)	6.15 (5.23–7.24)	314.4 (0.035)	1.39 (1.16–1.66)	1.46 (1.21–1.74)	1.43 (1.19–1.72)	
CHF (Yes:No)	11.8 (10.39–13.4)	963.4 (0.105)	3.07 (2.64–3.57)	2.54 (2.17–2.96)	2.55 (2.19–2.97)	
COPD (Yes:No)	4.83 (4.28–5.45)	523.7 (0.058)	—	1.37 (1.20–1.58)	1.37 (1.19–1.57)	
***OSA-related variables***						
Symptoms						
DS (Yes:No)	1.6 (1.41–1.81)	67.0 (0.008)	—	1.15 (1.02–1.29)	1.13 (1.01–1.28)	
PSG indexes						
AHI, total, events/hr (35:6.3)	1.49 (1.42–1.57)	192.5 (0.022)	—	—	—	
AWK, number (35:18)	1.35 (1.31–1.39)	243.6 (0.027)	—	1.07 (1.03–1.11)	1.06 (1.02–1.10)	
TST90SaO_2_, minutes (9:0)	1.06 (1.05–1.07)2.99 (2.53–3.54)^a^	250.7 (0.028)	—	1.02 (1.02–1.03)	1.50 (1.25–1.79)	
PLMI, events/hr (13.4:0.0)	1.18 (1.17–1.20)	232.2 (0.026)	—	1.05 (1.03–1.07)	1.05 (1.03–1.07)	
TST, hours (4.9:6.4)	1.8 (1.7–1.9)	366.1 (0.041)	—	1.19 (1.11–1.27)	1.20 (1.12–1.27)	
Heart rate, mean/TST, bpm (70:57)	1.38 (1.29–1.48)	69.9 (0.008)	—	1.30 (1.21–1.40)	1.28 (1.19–1.37)	
**LR χ^2^ (df)**	—	—	2,025.15 (10)	2,227.6 (16)	2,246.0 (18)	
**Bootstrap-corrected R^2^**	—	—	0.209	0.224	0.226	
**Bootstrap-corrected Harrell's C-index**	—	—	0.843	0.856	0.857	

For a unified presentation of all results and figures, the findings shown are for a single imputed data set (the third data set). For OSA-related variables, HRs and 95% CIs in this data set were all within 2% of coefficients pooled across five imputed data sets.

a TST90SaO_2_ variable was transformed using restricted cubic spline transformation with 4 knots at 0.5, 4, 9, and 100 minutes.

AWK, total number of awakenings in TST; bpm, beats per minute; df, degree of freedom; HR, hazard ratio; LR, likelihood ratio; PLMI, periodic limb movement index; TST90SaO_2_, sleep time spent with SaO_2_ less than 90%.

All models were well calibrated (all observed and predicted five-year survival within 5%) (Figure S2) and validated (optimism<0.004 for final model R^2^). The fit for the model with only traditional CV risk factors was statistically significantly worse than the final model (χ^2^ (8) = 220; *p*<0.0001), indicating that OSA-related predictors independently contribute to the development of the composite outcome.

### Nomogram

**Figure 6:**
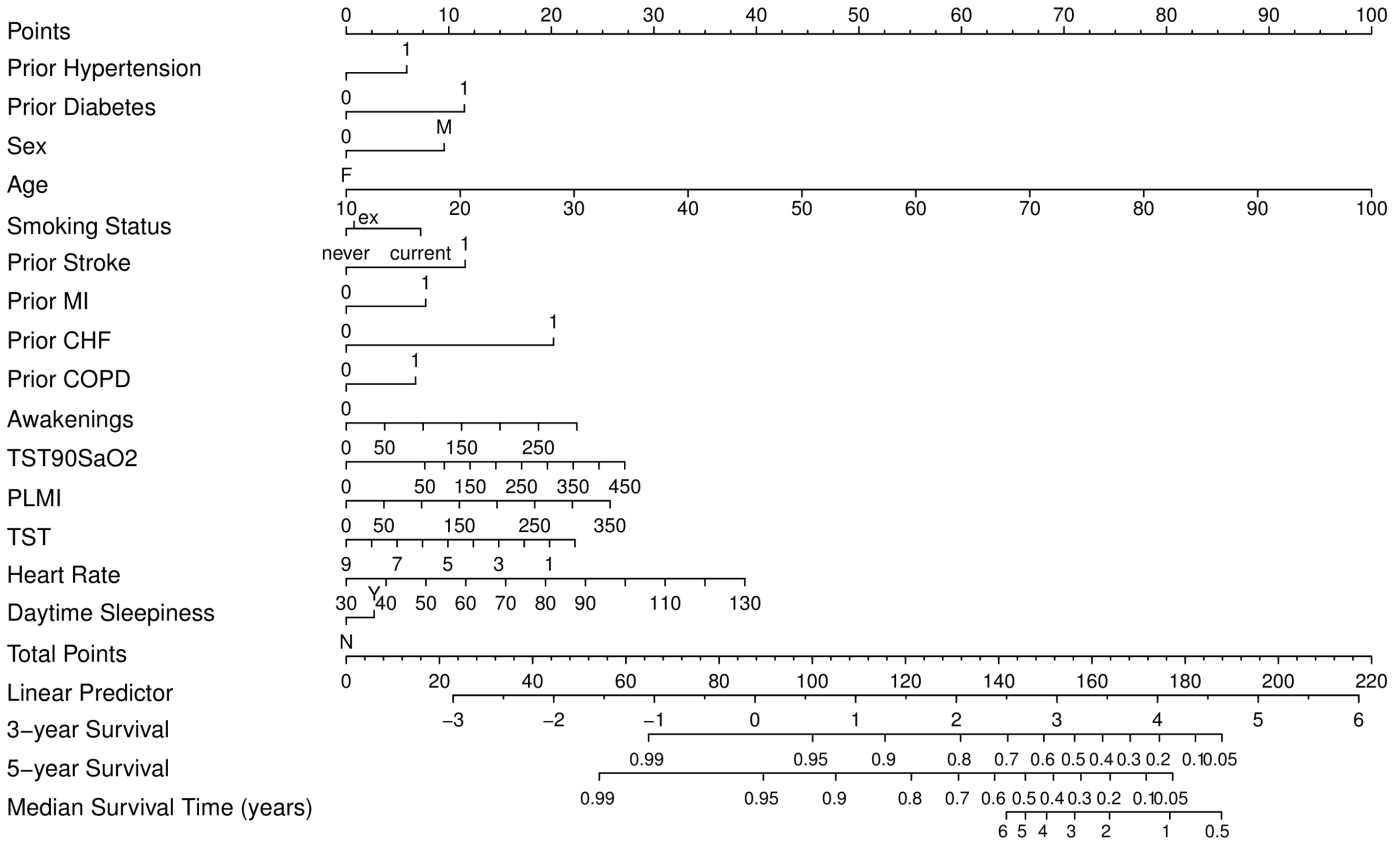
Clinical nomogram for obstructive sleep apnea patients. To obtain the nomogram predicted probability of three- and five-year event-free survival and to estimate median event-free survival, locate patient values at each axis, then draw a vertical line to the “Point” scale (axis) to determine how many points are attributed for each predictor. Sum the points for all predictors. Locate the sum on the “Total Points” scale. Draw a vertical line towards the “3-year Survival,” “5-year Survival,” and “Median Survival Time” axes to determine the three-year composite CV outcome-free survival, the five-year event-free survival, and to estimate median survival respectively. PLMI, periodic limb movement index; TST90SaO_2_, sleep time spent with SaO_2_ less than 90%.

Clinical nomograms estimating median survival time and the probability of 3 and 5 years event-free survival were based on the final transformed model (Figure 6). Variables with the widest point range in the nomogram provide the greatest discrimination. The points assigned to each predictor are presented in Table S3. To estimate the role of OSA-related variables using the nomogram, as an example we compared two different patients (Table 3). First, the total number of points was calculated for a relatively healthy person: male, 50 years old without signs of OSA and without comorbidities, never smoked, with mean heart rate of 70, and 7 hours of sleep. The total number of points for such a person is 75, indicating 5-year event-free survival of 0.95–0.99. The total number of points for a person with the same characteristics except with moderate-severe OSA is 99, indicating 5-year event-free survival of 0.95. The 24-point difference between the first and second person due to OSA-related predictors leads to a relatively small difference in 5-year event-free survival. However, the total number of points for a person of the same age, gender, and sleep time with comorbid illnesses, who smoked, and had a mean heart rate of 80 is 150, indicating 5-year event-free survival of 0.4–0.5 and median survival time of 4 years. For this person, a 24-point difference (attributed to OSA-related predictors) gives a total of 174, which corresponds to a 5-year event-free survival of about 0.1 and median survival time between 1 and 2 years, a clinically significant decrease.

**Table 3:** Example of clinical nomogram (point system) usage.

Characteristics	Person 1		Person 2		Person 3		Person 4	
	Value	Points	Value	Points	Value	Points	Value	Points
Sex	male	**10**	male	**10**	male	**10**	male	**10**
Age, years old	50	**44**	50	**44**	50	**44**	50	**44**
Smoking status	never	**0**	never	**0**	current	**7**	current	**7**
Prior HTN	No	**0**	No	**0**	Yes	**6**	Yes	**6**
Prior MI	No	**0**	No	**0**	Yes	**8**	Yes	**8**
Prior stroke	No	**0**	No	**0**	Yes	**12**	Yes	**12**
Prior CHF	No	**0**	No	**0**	Yes	**20**	Yes	**20**
Prior diabetes	No	**0**	No	**0**	Yes	**12**	Yes	**12**
Prior COPD	No	**0**	No	**0**	Yes	**7**	Yes	**7**
Mean heart rate, bpm	70	**16**	70	**16**	80	**19**	80	**19**
TST, hours	7	**5**	5	**10**	7	**5**	5	**10**
Presence of DS	No	**0**	Yes	**3**	No	**0**	Yes	**3**
AWK per TST	0	**0**	50	**4**	0	**0**	50	**4**
TST90SaO_2_, min	0	**0**	50	**8**	0	**0**	50	**8**
PLMI, per hr	0	**0**	50	**4**	0	**0**	50	**4**
Total number of points^a^	—	**75**	—	**99**	—	**150**	—	**174**
Proportion with 5-year event-free survival^b^	—	**0.95–0.99**	—	**0.95**	—	**0.4–0.5**	—	**0.1**

Person 1, male, 50 years old without signs of OSA and without comorbidities, never smoked, with mean heart rate of 70 and 7 hours of TST; Person 2, person 1+ moderate-severe OSA; Person 3, male, 50 years old with comorbidities, smoked, mean heart rate of 80 and 7 hours of TST; Person 4, person 3+ moderate-severe OSA.

a The sum of points attributable for each patient's characteristic.

b Corresponds to the total number of points.

AWK, total number of awakenings in TST; bpm, beats per minute; PLMI, periodic limb movement index; TST90SaO_2_, sleep time spent with SaO_2_ less than 90%.

### Separate Components of Composite CV Outcome

When the final model was applied to each component event, OSA-related variables were predictive of all-cause mortality (762 events), hospitalization for CHF (414 events), and stroke (100 events), but not for MI (145 events) (Table 4).

**Table 4:** Final model re-fitted for separate components of cardiovascular composite outcome (*n* = 10,149).

Variable (X:Y) Indicates the Two Values Being Compared	All-Cause Mortality (*n* events = 762)	MI (*n* events = 145)	Stroke (*n* events = 100)	CHF (*n* events = 414)
**Demographic characteristics**				
Sex (M:F)	1.38 (1.17–1.64)^a^	1.86 (1.23–2.82)^a^	1.63 (1.01–2.64)^a^	1.12 (0.89–1.42)
Age, years (59:40)	3.50 (3.04–4.02)^a^	2.57 (1.88–3.52)^a^	3.03 (2.07–4.44)^a^	2.44 (2.00–2.98)^a^
**History**				
Smoking Status				
Ex-smoker:never	1.03 (0.87–1.22)	0.91 (0.61–1.36)	0.84 (0.52–1.35)	0.99 (0.79–1.25)
Current:never	1.52 (1.24–1.86)^a^	1.60 (1.03–2.48)^a^	1.56 (0.90–2.72)	1.39 (1.04–1.85)^a^
***Prior comorbidities***				
HTN (Yes:No)	1.22 (1.03–1.44)^a^	1.61 (1.09–2.38)^a^	2.24 (1.35–3.70)^a^	1.52 (1.18–1.94)^a^
Diabetes (Yes:No)	1.67 (1.42–1.96)^a^	2.43 (1.69–3.50)^a^	1.52 (0.98–2.38)	1.83 (1.48–2.26)^a^
Stroke (Yes:No)	1.57 (1.18–2.07)^a^	1.39 (0.67–2.88)	3.18 (1.74–5.82)^a^	1.56 (1.11–2.20)^a^
MI (Yes:No)	1.27 (1.01–1.59)^a^	2.44 (1.50–3.98)^a^	1.00 (0.51–1.93)	1.39 (1.07–1.79)^a^
CHF (Yes:No)	2.12 (1.76–2.55)^a^	0.95 (0.57–1.57)	1.24 (0.72–2.15)	6.56 (5.15–8.37)^a^
COPD (Yes:No)	1.53 (1.30–1.81)^a^	1.34 (0.90–2.01)	1.31 (0.82–2.08)	1.46 (1.16–1.83)^a^
**OSA-related variables**				
**Symptoms**				
DS (Yes:No)	1.05 (0.91–1.22)	1.15 (0.83–1.61)	1.46 (0.97–2.19)	1.19 (0.98–1.46)
**PSG indexes**				
AWK, number (35:18)	1.06 (1.02–1.11)^a^	0.96 (0.85–1.09)	1.14 (1.02–1.27)^a^	1.08 (1.02–1.15)^a^
TST90SaO_2_, minutes (9:0)^b^	1.58 (1.26–1.98)^a^	1.31 (0.79–2.18)	1.24 (0.68–2.25)	1.92 (1.39–2.64)^a^
PLMI, events/hr (13.4:0.0)	1.05 (1.02–1.07)^a^	0.98 (0.91–1.05)	1.01 (0.94–1.09)	1.05 (1.02–1.09)^a^
TST, hours (4.9:6.4)	1.16 (1.07–1.27)^a^	1.05 (0.86–1.28)	1.54 (1.23–1.93)^a^	1.28 (1.14–1.43)^a^
Heart rate, mean/TST, bpm (70:57)	1.39 (1.28–1.52)^a^	0.92 (0.74–1.15)	0.96 (0.74–1.24)	1.22 (1.08–1.37)^a^
**LR χ^2^ (df)**	1,673.95 (18)	210.03 (18)	218.30 (18)	1,371.66 (18)
**R^2^**	0.21	0.09	0.14	0.25

a Significant association (*p* < 0.05).

b TST90SaO_2_ was transformed using a restricted cubic spline with 4 knots at 0.5, 4, 9, and 100 minutes.

AWK, total number of awakenings in TST; bpm, beats per minute; df, degree of freedom; LR, likelihood ratio; PLMI, periodic limb movement index; TST90SaO_2_, sleep time spent with SaO_2_ less than 90%.

### Interactions

The association between TST90SaO_2_ (9 versus 0 minutes) and the composite outcome was significantly (*p* = 0.04) stronger for females (HR = 2.21, 95% CI 1.56–3.11) than for males (HR = 1.29, 95% CI 1.05–1.57).

### CPAP Treatment

Among 4,733 individuals who underwent a diagnostic sleep study between 2004 and 2010, 762 (16%) submitted a CPAP claim and 333 (7%) experienced a composite event. In our final model, a CPAP claim was not associated with risk of an event (HR = 0.85, 95% CI 0.64–1.14, *p* = 0.3). To assess the final model on an untreated sample, patients were censored at the time of a CPAP claim; all predictors except DS remained significantly associated with outcome (Table 5).

**Table 5:** Association between OSA-related variables and the composite CV outcome on untreated subsample versus entire cohort.

Variable (X:Y) Indicates the Two Values Being Compared	Untreated Subsample^a^ (≥2,004) (*n* Total = 4,733, *n* Events = 270)	For Entire Cohort (*n* Total = 10,149, *n* Events = 1,172)
**Demographic characteristics**		
Sex (M:F)	1.34 (1.01–1.78)	1.55 (1.35–1.79)
Age, years (59:40)	2.48 (1.97–3.13)	2.66 (2.38–2.97)
**History**		
**Smoking Status**		
Ex-smoker:never	1.10 (0.83–1.46)	1.04 (0.90–1.19)
Current:never	1.61 (1.15–2.26)	1.40 (1.19–1.65)
***Prior comorbidities***		
HTN (Yes:No)	1.72 (1.25–2.36)	1.31 (1.15–1.51)
Diabetes (Yes:No)	1.36 (1.03–1.79)	1.71 (1.50–1.94)
Stroke (Yes:No)	1.17 (0.70–1.93)	1.71 (1.36–2.15)
MI (Yes:No)	1.46 (1.04–2.06)	1.43 (1.19–1.72)
CHF (Yes:No)	3.03 (2.23–4.12)	2.55 (2.19–2.97)
COPD (Yes:No)	1.13 (0.85–1.51)	1.37 (1.19–1.57)
**OSA-related variables**		
**Symptoms**		
DS (Yes:No)	1.12 (0.88–1.44)	1.13 (1.01–1.28)
**PSG indexes**		
AWK, number (35:18)	1.17 (1.09–1.26)	1.06 (1.02–1.10)
TST90SaO_2_, minutes (9:0)^b^	1.70 (1.18–2.47)	1.50 (1.25–1.79)
PLMI, events/h (13.4:0.0)	1.07 (1.02–1.11)	1.05 (1.03–1.07)
TST, hours (4.9:6.4)	1.22 (1.06–1.39)	1.20 (1.12–1.27)
Heart rate, mean/TST, bpm (70:57)	1.22 (1.05–1.43)	1.28 (1.19–1.37)

a Subcohort of patients who underwent a diagnostic sleep study since 2004; patients who claimed CPAP through ADP data set before event of interested occurred were censored at the time of a CPAP claim.

b TST90SaO_2_ was transformed using a restricted cubic spline with 4 knots at 0.5, 4, 9, and 100 minutes.

AWK, total number of awakenings in TST; bpm, beats per minute; PLMI, periodic limb movement index; TST90SaO_2_, sleep time spent with SaO_2_ less than 90%.

## Discussion

In a large clinical cohort, 11.5% of individuals experienced a composite CV outcome over a median 68 months of follow-up. Our incidence rate of 2 per 100 person-years was similar to rates reported in other clinically-based sleep studies [27],[28]. While AHI was found to predict CV events in a univariate analysis, there was no significant association in a multivariable model adjusted for potential confounders. In contrast, the multivariable models identified other OSA-related factors as independent and significant predictors of the occurrence of CV events and all-cause mortality. The results obtained were driven by all-cause mortality, hospitalized heart failure and stroke, and were replicated on a subsample of untreated patients. Using a nomogram, we demonstrated that OSA-related variables independently contribute to predicted three- and five-year event-free survival and median survival time, over and above traditional risk factors. Though the difference attributed to OSA-related predictors for a relatively healthy person seems small, for individuals with high baseline risk, even a small increase in risk due to OSA will place a patient in considerably higher risk group.

The significant association between AHI and the composite CV outcome in our univariate analyses is consistent with findings of other studies. However, the persistent significance of this association in multivariable models in other studies, but not in ours, could be explained by methodological differences between our study and other studies. For example, large, community-based studies may not include potentially more important OSA-related predictors or selectively report findings from subgroup analyses [13].

Variations in the definition of hypopnea may be important. We used a definition of hypopnea that does not demand the occurrence of oxygen desaturation. The ability of AHI to predict CV disease was found to improve if 3% or 4% oxygen desaturation is required for the scoring of hypopneas [29], indicating the probable importance of hypoxia in mediating CV risk from OSA and raising the question of whether a more direct measure of hypoxia might be more predictive than AHI.

In fact, the strongest OSA-related predictor of CV events in our study was the sleep time spent with SaO_2_<90%. The association between TST90SaO_2_ and the outcome of interest remains significant for both sexes, and across a spectrum of age, BMI, DS, and comorbid CV diseases. Our finding that a measure of hypoxia predicts CV risk is consistent with emerging evidence in both animal models and humans that intermittent hypoxia may be a crucial mechanistic link whereby OSA causes oxidative stress, metabolic derangement, and endothelial damage [30],[31].

The remainder of the OSA-related variables predictive of CV risk were mainly reflective of either sleep fragmentation or sympathetic activity. Increases in the number of awakenings or periodic leg movements or decreases in TST may represent the direct effects of OSA on sleep fragmentation and deprivation that can increase risk by inducing endothelial dysfunction and sympathetic activation [32]. Despite limited evidence elsewhere on the association between the number of awakenings and CV events, the evidence is growing regarding the relationship of PLM to CV events [7],[33],[34]. Low TST may indicate insomnia, which by itself can lead to increased risk of all-cause mortality and development of CV events [35]. However, in patients with chronic insomnia, most awakenings may be caused by sleep-disordered breathing [36]. Many of the CV consequences of OSA may be mediated by activation of sympathetic activity, and represented by increased heart rate [5]. Finally, available data suggest an association between mortality (all-cause and from CV disease) and excessive DS [37].

Despite strong evidence from randomized controlled trials of a causal relationship between OSA and markers of risk of CV events, it remains to be proven whether OSA-induced HTN translates into increased morbidity and mortality [38]. A recent randomized controlled trial found that in patients with OSA without DS, the prescription of CPAP did not result in a statistically significant reduction in the incidence of a combined outcome of HTN or CV events, compared with usual care [39]. In the same trial, OSA severity, assessed by the AHI and sleep time spent with SaO_2_ less than 90%, was not related to the combined outcome. However, in a *post hoc* analysis, patients with worse oxygen desaturation at night and with CPAP adherence of less than four hours per night showed a higher rate of HTN or CV events than the control group.

Although evidence has been inconsistent on the association between OSA-related predictors and CV events in females and elderly individuals [7], we found that the significant association extended to these groups, in part due to the composition of our cohort. Our cohort included a large number of women over 50 years of age, who we assume are postmenopausal and therefore at increased risk of CV events [40],[41].

Our research addresses many limitations of previously published observational studies. Our study includes over 10,000 patients from a clinically-based sleep cohort and consequently has a large number of events, allowing us to assess many variables in the model and control for all available confounders. Data were consistently collected and used the same PSG scoring criteria over time. We included patients with a wide range of OSA severity and a relatively large number of females. We matched a high percentage of patients (89%) to administrative data, had long and complete follow-up through health administrative data, used validated algorithms to define CV outcomes and comorbidities at baseline, and, finally, used rigorous methods for missing data, model selection, calibration, and validation. The format of the nomogram used as a predictive tool allows predictions based on any combination of patient characteristics, not only from categorical, but also from continuous (possibly transformed) variables and interactions between variables. The nomogram allows the use of a complicated model in clinical prediction, improving accuracy of predictions over simpler points-based scores that require categorization of risk using arbitrary categories [42],[43].

As with any observational study, there are limitations related to availability of data. Some important confounders (e.g., level of cholesterol, race, treatment of hypertension) were not available. However, our model with CV risk factors had high predictive and discriminative ability, reflecting that the majority of important predictors were included. Assessing the sensitivity of obtained results to unmeasured confounders using approach recommended by Lin and colleagues [44], we found that unmeasured confounders (e.g., treatment for hypertension) should be really strong (i.e., increased both the hazard of composite CV outcome and the probability of severe SaO_2_ [75th percentile] 4-fold) to change the association between OSA and time spent with SaO_2_ less than 90% to non-significant. Our findings are based on patients referred to a single centre, which may reduce the generalizability of our findings. Validation of our results in other patient populations is recommended. Furthermore, there is no accepted definition of intermittent hypoxemia that includes both SaO_2_ variability and severity. Although oxygen desaturation index (ODI), the hourly average number of desaturation episodes, could be a useful predictor, it was not available in our study. However, while ODI reflects frequency of oxygen desaturation, time spent with SaO_2_<90% reflects its severity. The non-significant relationship between treatment and development of composite CV outcome that we found in the post-2004 cohort could be explained by lack of information about CPAP adherence, treatment approaches other than CPAP, and reduced power in this analysis. Moreover, the association between CPAP treatment and CV events can be attenuated because some patients were treated unnecessarily. Finally, it is naive to assume that all predictor levels will remain constant during the follow-up time [45]. Their trajectories (e.g., weight gain, OSA progression over time) were not known for our patients and not assessable at baseline when risk prediction was made.

Though we have developed a nomogram for prediction to show how the baseline predictors relate to 3- and 5-year outcomes, we cannot strongly recommend its use in a clinical setting before it has undergone external validation. Rather, we used the nomogram to show the strength of the various factors on absolute risks of the composite CV outcome. Further, although the increments observed in c-statistics between final model and CV risk factors were small (0.857–0.843 = 0.014), it has been shown that the increase in c-statistics is very small when the baseline model's c-index is large: “good models are harder to improve upon” [46],[47].

We showed that OSA-related factors other than AHI are important predictors of the composite CV outcome. We believe a revision of the operative definition of OSA may be necessary, to reflect not simply the frequency of apneas and hypopneas, but the actual physiologic consequences that result—the severity of oxygen desaturation, sleep fragmentation, sleep deprivation, and sympathetic activation. It is these “downstream” phenomena that we have found to be more predictive of CV risk. The OSA-related predictors identified in our study could be collected using more limited recordings than PSG, potentially in the home setting.

### Conclusions

AHI was significantly associated with a composite CV outcome in univariate analyses; however, this association became non-significant after controlling for potential confounders. Other OSA-related predictors, such as sleep time spent with SaO_2_ less than 90%, the number of awakenings, mean heart rate, TST, or presence of excessive DS, were significantly and independently associated with a 5% to 50% increased risk of development of the composite CV outcome even after controlling for known CV risk factors.

## Supporting Information

Alternative Language Abstract S1 **Spanish translation of the abstract.** Translation by Romina Brignardello-Petersen.
(DOCX)

Alternative Language Abstract S2 **Russian translation of the abstract.** Translation by Tetyana Kendzerska.
(DOCX)

Figure S1 **Effect of sleep time spent with SaO_2_ less than 90%, on the log hazard of composite CV outcome.** A restricted cubic spline transformation with 4 knots was used to model the non-linearity in this relationship. The shaded area is a 95% confidence band.
(TIF)

Figure S2 **Calibration plot of the final model (predicted versus observed five-year survival).** All observed and predicted 5-year survival values were within 5%. The final model was well calibrated: for 14 of 17 groups by 500 patients prediction was good. X, resampling optimism added, B = 150; Based on observed–predicted. Each group is 500 individuals; gray is ideal.
(TIF)

Table S1 **List of variables collected in sleep laboratory (each patient in the cohort underwent an overnight full standard PSG recording which was scored manually by a sleep technologist and reviewed by a board-certified sleep physician).** *These variables were excluded from the main analyses as more than 50% of individuals had missing values.
(DOCX)

Table S2 **Information about variables derived from administrative data.** List of data sets used: The Registered Persons Database (RPDB); Canadian Institute for Health Information Discharge Abstract database (CIHI-DAD) and the Same Day Surgery (CIHI-SDS); Ontario Health Insurance Plan Physician Services Claims database (OHIP); NACRS (National Ambulatory Care Reporting System); the Ontario Diabetes Database (ODD); HYPERTENSION; Ontario Congestive Heart Failure database (CHF); Ontario Chronic Obstructive Pulmonary Disease database; Ontario Mental Health Reporting System (OMHRS) stand-alone admissions data set; Ontario Cancer Registry Data (OCRD); Ontario Registrar General Death (ORGD) data; Assistive Devices Program data set (ADP). CABG, coronary artery bypass graft surgery; PCI, percutaneous coronary intervention. *OHIP diagnostic codes except written OHIP fee codes.
(DOCX)

Table S3 **A simple points system based on the beta-coefficients from the final transformed model.** Points per predictors, total points and corresponding three- and five-year event-free survival and median survival time are presented. Points per unit of linear predictor: 21.59161. Linear predictor units per point: 0.04631428.
(DOCX)

## Abbreviations

ADP: Assistive Devices Program
AHI: apnea-hypopnea index
BMI: body mass index
CHF: congestive heart failure
COPD: chronic obstructive pulmonary disease
CPAP: continuous positive airway pressure
CV: cardiovascular
DS: daytime sleepiness
HR: hazard ration
HTN: hypertension
MI: myocardial infarction
OSA: obstructive sleep apnea
PSG: polysomnographical
SaO_2_: oxygen saturation
TST: total sleep time
TST90SaO_2_: sleep time spent with SaO_2_<90%
