# Supplementary material for: Obstructive Sleep Apnea and Risk of Cardiovascular Events and All-Cause Mortality: A Decade-Long Historical Cohort Study
Source: PLoS Med. 2014 Feb 4;11(2):e1001599. doi: 10.1371/journal.pmed.1001599 (PMC3913558; doi:10.1371/journal.pmed.1001599)
Supplement: Alternative Language Abstract S1 — Spanish translation of the abstract. Translation by Romina Brignardello-Petersen. (DOCX) [file pmed.1001599.s001.docx]

**Language: Spanish**

**Translator : Romina Brignardello-Petersen**

Institute of Health Policy, Management and Evaluation,

Faculty of Medicine, University of Toronto, Toronto, Canada

Evidence-Based Dentistry Unit, Faculty of Dentistry, University of Chile, Santiago, Chile

**Título**: Síndrome de apnea obstructiva durante el sueño y el riesgo de eventos cardiovasculares y la mortalidad por todas las causas: un estudio de cohortes histórico de una década
**Abstracto**
Antecedentes: La apnea obstructiva durante el sueño (AOS) ha sido descrita como un factor de riesgo para enfermedad cardiovascular (ECV). Aunque el índice de apnea-hipopnea (IAH) es la medida más utilizada de la AOS, otras variables menos estudiadas relacionadas con AOS pueden ser más relevantes patofisiológicamente y ofrecer una mejor predicción. El objetivo de este estudio fue evaluar la relación entre las variables relacionadas con AOS y el riesgo de eventos ECV.
Métodos y resultados: se realizó un estudio de cohortes histórico utilizando una base de datos clínicos y datos de salud administrativa, en donde se incluyó a adultos referidos por sospecha de AOS que fueron sometidos a polisomnografía diagnóstica en el laboratorio del sueño del Hospital St Michael (Toronto , Canadá ) entre 1994 y 2010. El seguimiento de los pacientes fue guardado entre los datos administrativos del Instituto de Ciencias Clínicas de Evaluación (Ontario , Canadá) hasta Mayo de 2011 para determinar la ocurrencia de un desenlace compuesto (infarto de miocardio, accidente cerebrovascular, insuficiencia cardíaca congestiva, procedimientos de revascularización o muerte por todas otras causas). Los modelos de regresión de Cox fueron utilizados para investigar la asociación entre las variables relacionadas con OSA y el desenlace compuesto controlando por factores de riesgo comunes. Los resultados se expresaron como razón de riesgo (RR) e IC del 95%; para las variables continuas, los RR comparan los porcentajes de 75 y 25.
Para un periodo promedio de seguimiento de 68 meses, 1.172 (11,5 %) de los 10.149 participantes experimentaron un desenlace compuesto. En un modelo que ajustó con todos los factores, excepto por las variables relacionadas con AOS e IAH, fueron predictores independientes significativos: el tiempo con saturación de oxígeno < 90 % (9 minutos o bien 0; HR = 1.5, IC del 95 %: 1.25 a 1.79), el tiempo de sueño ( 4.9 o bien 6.4 horas; RR = 1.2, IC 95 %: 1.12 a 1.27) , número de veces que la persona se despertó (35 o 18; RR = 1.06, IC 95 %: 1.2 a 1.10) , movimientos periódicos de las piernas (13 o bien 0 por hora; RR = 1.05, IC 95%: 01.03 a 01.07), frecuencia cardíaca (70 o bien 56 lpm; RR = 1.28, IC del 95 %: 1.19 a 1.37), y somnolencia diurna (RR = 1.13, IC 95%: 1.1 a 1.28).
La principal limitación del estudio fue la falta de información sobre la adherencia al CPAP.
Conclusión: Los factores relacionados con AOS, distintos de IAH, mostraron ser predictores importantes del desenlace compuesto CV y deben ser considerados en futuros estudios y la práctica clínica.
