# Supplementary material for: Obstructive Sleep Apnea and Risk of Cardiovascular Events and All-Cause Mortality: A Decade-Long Historical Cohort Study
Source: PLoS Med. 2014 Feb 4;11(2):e1001599. doi: 10.1371/journal.pmed.1001599 (PMC3913558; doi:10.1371/journal.pmed.1001599)
Supplement: Alternative Language Abstract S2 — Russian translation of the abstract. Translation by Tetyana Kendzerska. (DOCX) [file pmed.1001599.s002.docx]

**Language: Russian**

**Translator: Tetyana Kendzerska**

**Название статьи: Обструктивное апноэ сна и риск развития сердечно-сосудистых заболеваний и общей смертности: 10-летнее историческое когортное исследование.**

**Абстракт**

**Вступление:** Существуют данные свидетельствуют о том, что обструктивное апноэ сна (ОАС) может выступать фактором риска развития сердечно-сосудистых заболеваний. Несмотря на то, что индекс апноэ-гипопноэ (ИАГ) является самым широкого используемым критерием ОАС, другие менее изученные ОАС-зависимые переменные могут быть более релевантными патофизиологически и обеспечивать лучшее прогнозирование. Целью данного исследования является оценка взаимосвязи между ОАС-зависимыми переменными и риском развития сердечно-сосудистых заболеваний.

**Методы и результаты:** Данное когортное исследование было основано на госпитальной клинической базе данных, и административных данных системы здравоохранения. Взрослые, диагностированные с подозрением на ОАС и прошедшие диагностическую полисомнографию в лаборатории сна госпиталя St Michael’s (Торонто, Канада) в период между 1994-м и 2010-м годами, были отслежены в административной базе данных Института Научных Клинических Исследований (Онтарио, Канада) до мая 2011 года с целью изучить возникновение комбинированных клинических исходов (инфаркт миокарда, инсульт, хроническая сердечная недостаточность, реваскуляризация, или смерть от любой причины). Для оценки связи между основными ОАС-зависимыми переменными и комбинированными клиническими исходами с контролем традиционных факторов риска были использованы регрессионные модели Кокса. Результаты были представлены как отношение рисков (ОР) и 95% доверительный интервал (ДИ); для непрерывных переменных, ОР было представлено для 75-го и 25-го процентилей.

Медиана времени наблюдения пациентов составила 68 месяцев. В течение этого времени у 1172 (11,5%) из 10 149 участников мы наблюдали развитие комбинированных клинических исходов. В полностью скорректированной модели, следующие ОАС-зависимые переменные были значимыми независимыми предикторами: время сна с уровнем кислорода < 90% (9 минут в сравнении с 0; ОР=1.5, 95% ДИ: 1.25-1.79), время сна (4.9 часов против 6.4 часа; ОР =1.2, 95% ДИ: 1.12-1.27), количество ночных пробуждений (35 в сравнении с 18 раз в течение сна; ОР =1.06, 95% ДИ: 1.02-1.10), периодические движения ног (13 против 0 в час; ОР =1.05, 95% ДИ: 1.03-1.07), сердцебиение (70 против 56 ударов в минуту; ОР =1.28, 95% ДИ: 1.19-1.37), и наличие дневной сонливости (ОР =1.13, 95% ДИ: 1.01-1.28).

Основным ограничением данного исследования был недостаток информации о соблюдение режима лечения.

**Выводы:**: ОАС-зависимые факторы за исключением ИАГ являются важными предикторами комбинированных сердечно-сосудистых исходов и должны быть приняты во внимание в будущих исследования и клинической практике.
