# Supplementary figures and images for: Obstructive Sleep Apnea and Risk of Cardiovascular Events and All-Cause Mortality: A Decade-Long Historical Cohort Study
Source: PLoS Med. 2014 Feb 4;11(2):e1001599. doi: 10.1371/journal.pmed.1001599 (PMC3913558; doi:10.1371/journal.pmed.1001599)

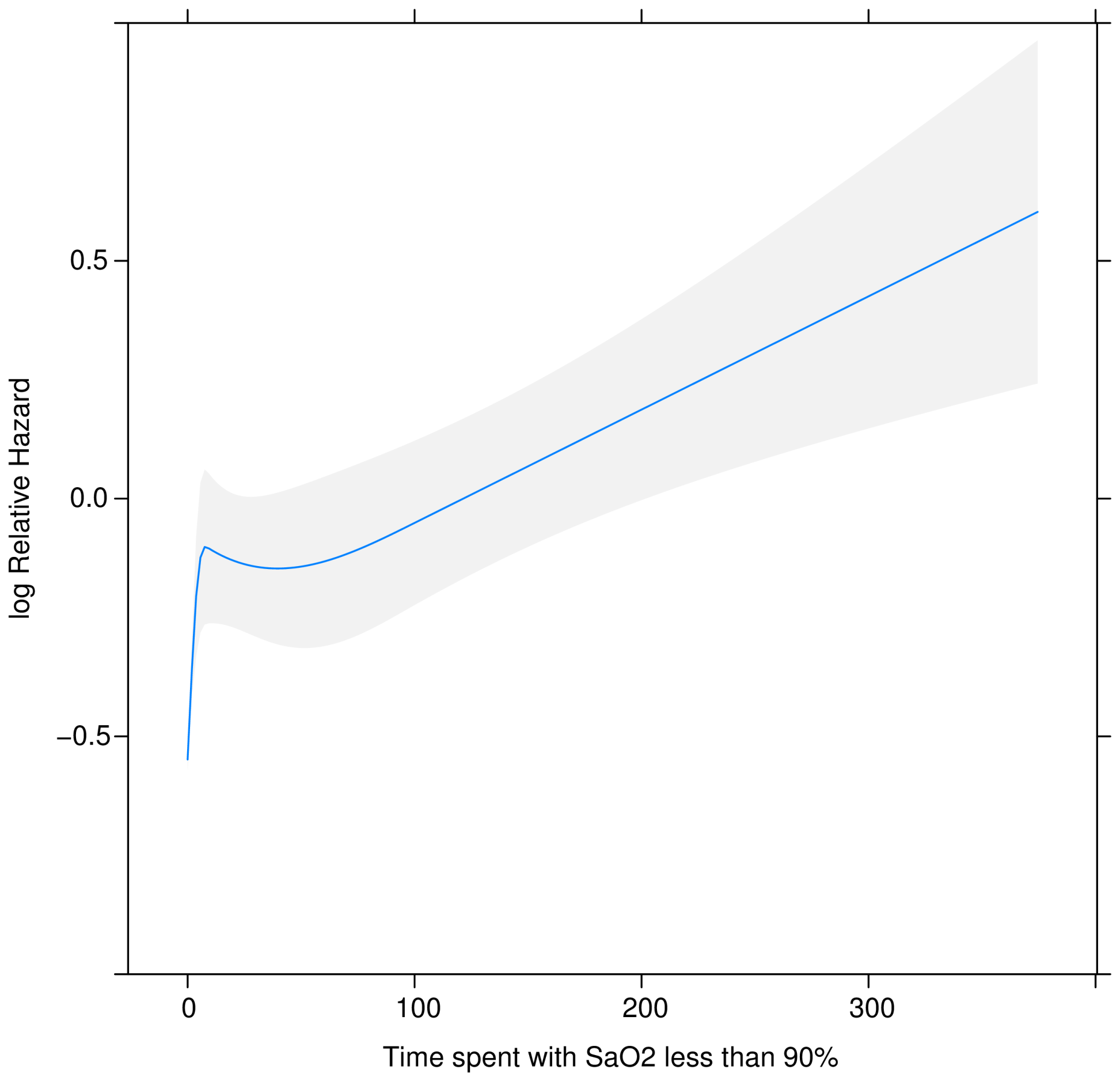

Supplement: Figure S1 — Effect of sleep time spent with SaO2 less than 90%, on the log hazard of composite CV outcome. A restricted cubic spline transformation with 4 knots was used to model the non-linearity in this relationship. The shaded area is a 95% confidence band. (TIF) [file pmed.1001599.s003.tif]

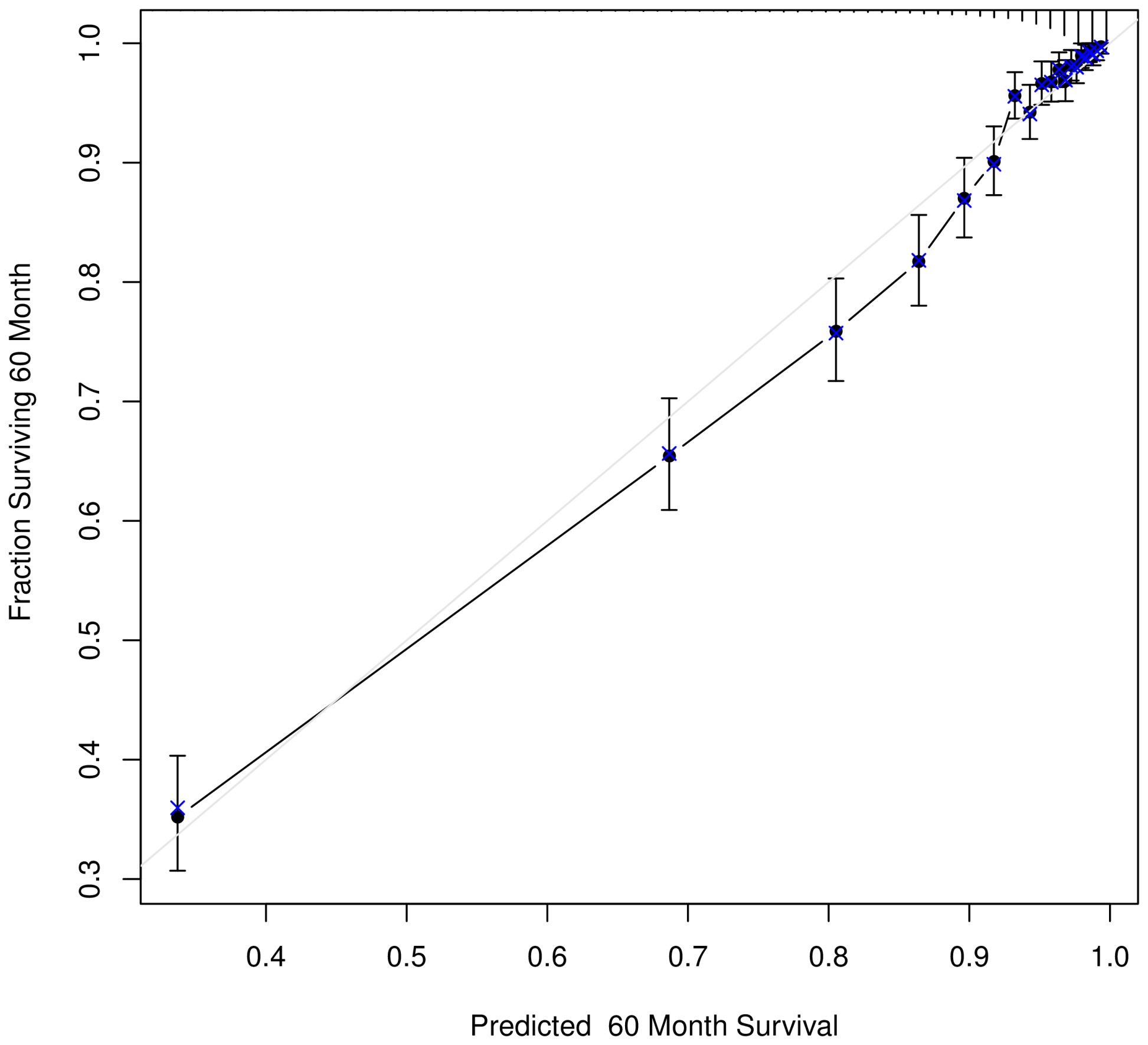

Supplement: Figure S2 — Calibration plot of the final model (predicted versus observed five-year survival). All observed and predicted 5-year survival values were within 5%. The final model was well calibrated: for 14 of 17 groups by 500 patients prediction was good. X, resampling optimism added, B = 150; Based on observed–predicted. Each group is 500 individuals; gray is ideal. (TIF) [file pmed.1001599.s004.tif]
