# Supplementary material for: Obstructive Sleep Apnea and Risk of Cardiovascular Events and All-Cause Mortality: A Decade-Long Historical Cohort Study
Source: PLoS Med. 2014 Feb 4;11(2):e1001599. doi: 10.1371/journal.pmed.1001599 (PMC3913558; doi:10.1371/journal.pmed.1001599)
Supplement: Table S1 — List of variables collected in sleep laboratory (each patient in the cohort underwent an overnight full standard PSG recording which was scored manually by a sleep technologist and reviewed by a board-certified sleep physician). *These variables were excluded from the main analyses as more than 50% of individuals had missing values. (DOCX) [file pmed.1001599.s005.docx]

**Table S1**. List of variables collected in sleep laboratory (each patient in the cohort underwent an overnight full standard PSG recording which was scored manually by a sleep technologist and reviewed by a board-certiﬁed sleep physician).

| **Name** | **Description** | **Measurement** |
| --- | --- | --- |
| ***Demographic characteristics (self-reported by patients)*** | | |
| Sex |  | Male/Female |
| Age |  | years |
| ***Symptoms related to OSA (self-reported by patients)*** | | |
| *Daytime sleepiness* | | |
| Epworth Sleepiness Scale, total | Total score | ranged from 0 to 24 |
| Item #1 | Sitting and reading | 0 = would never doze  1 = slight chance of dozing  2 = moderate chance of dozing  3 = high chance of dozing |
| Item #2 | Watching TV | from 0 to 3 |
| Item #3 | Sitting inactive in a public place (e.g. a theatre or at a meeting) | from 0 to 3 |
| Item #4 | As a passenger in a car for an hour without a break | from 0 to 3 |
| Item #5 | Lying down in the afternoon when circumstances permit | from 0 to 3 |
| Item #6 | Sitting and talking to someone | from 0 to 3 |
| Item #7 | Sitting quietly after a lunch without alcohol | from 0 to 3 |
| Item #8 | In a car while stopped for a few minutes in traffic | from 0 to 3 |
| Self-reported DS | “During the day, do you ever fall asleep unintentionally?” | Yes/No |
| *Other symptoms* | | |
| Self-reported snoring | Do you snore? | Yes/No |
| Observed cessation of breathing | Has anyone ever told you that you stop breathing while you sleep? | Yes/No |
| Observed restless sleeper | Have you been told that you are a restless sleeper? | Yes/No |
| Wake unrefreshed | Do you feel refreshed when you wake up? | Yes/No |
| Self-reported morning headache | Do you often wake up with headaches in the morning? | Yes/No |
| ***History (self-reported by patients)*** | | |
| Smoking status |  | current  ex-smoker  never |
| Alcohol consumption* | "Y" > 7 alcoholic beverages per week, other - "N" | Yes/No |
| SR HiBP | Do you, or have you ever suffered from high blood pressure? | Yes/No |
| SR MI | Self-reported myocardial infarction | Yes/No |
| SR Stroke | Self-reported stroke | Yes/No |
| History of Family Snore | Does anybody else in your family snore loudly? | Yes/No |
| History of Family Apnea Diagnosis | Has anyone in your family been diagnosed with sleep apnea? | Yes/No |
| ***Physical examination by sleep technician according to the lab manual*** | | |
| BPSYSPM* | Systolic blood pressure, pm | mm Hg |
| BPDIAPM* | Diastolic blood pressure, pm | mm Hg |
| WGT | weight | kg |
| HGT | height | cm |
| NECK | neck circumference | cm |
| WAIST | waist circumference | cm |
| HIP | hip circumference | cm |
| ***PSG recording: PSG software used is different version of Sandman (current - 7.3)*** | | |
| TST | Time in Bed (TIB) – Sleep Latency (SL) | hours |
| Sleep efficiency | TST/TIB | % |
| STAGE1 PER | The PSG was scored manually for sleep stage according to established criteria using the EEC, EOG and EMG records [[1](#_ENREF_1)]. | % of stage 1 |
| STAGE2 PER |  | % of stage 2 |
| STAGE3 PER |  | % of stage 3 |
| STAGE4 PER |  | % of stage 4 |
| REM PER |  | % of REM |
| HRMEAN TST | Overall mean heart rate in TST | bpm |
| PLMI TST | The number of periodic leg movements per hour of TST | events/hr. |
| ArI, total | Total Arousals index in TST | events/hr. |
| AWK TST | Total Awakenings, number in TST | events/TST |
| MEANO2SAT_TST | Overall Mean SaO_2_% in TST | % |
| O2SAT90MIN_TST | Duration of SaO_2_<90% in TST | minutes |
| ProcSat_less90TST | % of SaO_2_<90%, in TST | % |
| DBMEAN_WAKE* | SNORING INTENSITY, mean during the wake to adjust for background noisy | DBMEAN_WAKE |
| *Apnea and hypopnea events* | | |
| Obstructive apnea/hypopnea event | Must fulfill criterion 1 or 2, plus criterion 3 of the following [[2](#_ENREF_2)]:  1. A clear decrease (>50%) from baseline in the amplitude of a valid measure of breathing during sleep. Baseline is defined as the mean amplitude of stable breathing and oxygenation in the two minutes preceding onset of the event (in individuals who have a stable breathing pattern during sleep) or the mean amplitude of the three largest breaths in the two minutes preceding onset of the event (in individuals without a stable breathing pattern).  2. A clear amplitude reduction of a validated measure of breathing during sleep that does not reach the above criterion but is associated with either an oxygen desaturation of >3% or an arousal.  3. The event lasts 10 seconds or longer | |
| ApnOI TST | Obstructive apnea Index in TST | events/hr. |
| ApnTotI TST | Total apnea Index in TST | events/hr. |
| HypOI TST | Obstructive hypopneas Index in TST | events/hr. |
| HypTotI TST | Total hypopneas Index in TST | events/hr. |
| AHIO TST | Obstructive apnea-hypopneas Index in TST | events/hr. |
| AHIO NREM | Obstructive apnea-hypopneas Index in NREM | events/hr. |
| AHIO REM | Obstructive apnea-hypopneas Index in REM | events/hr. |
| AHI TST | Total apnea-hypopneas Index in TST | events/hr. |
| AHITot NREM | Total apnea-hypopneas Index in NREM | events/hr. |
| AHITot REM | Total apnea-hypopneas Index in REM | events/hr. |
| AHDUR MEAN | Total apnea-hypopneas, Mean Duration | min |
| AHDUR MAX | Total apnea-hypopneas, Longest Event | min |

*** -** these variables were excluded from the main analyses as more than 50% of subjects had missing values

**References**

1. Rechtschaffen, A., Kale,s A, eds. *A manual of standardized terminology, techniques and scoring system of sleep stages in human subjects.* Los Angeles: Brain Information Service/Brain Research Institute, University of California, 1968.

2. *Sleep-related breathing disorders in adults: recommendations for syndrome definition and measurement techniques in clinical research. The Report of an American Academy of Sleep Medicine Task Force.* Sleep, 1999. **22**(5): p. 667-89.
