# Supplementary material for: Obstructive Sleep Apnea and Risk of Cardiovascular Events and All-Cause Mortality: A Decade-Long Historical Cohort Study
Source: PLoS Med. 2014 Feb 4;11(2):e1001599. doi: 10.1371/journal.pmed.1001599 (PMC3913558; doi:10.1371/journal.pmed.1001599)
Supplement: Table S2 — Information about variables derived from administrative data. List of data sets used: The Registered Persons Database (RPDB); Canadian Institute for Health Information Discharge Abstract database (CIHI-DAD) and the Same Day Surgery (CIHI-SDS); Ontario Health Insurance Plan Physician Services Claims database (OHIP); NACRS (National Ambulatory Care Reporting System); the Ontario Diabetes Database (ODD); HYPERTENSION; Ontario Congestive Heart Failure database (CHF); Ontario Chronic Obstructive Pulmonary Disease database; Ontario Mental Health Reporting System (OMHRS) stand-alone admissions data set; Ontario Cancer Registry Data (OCRD); Ontario Registrar General Death (ORGD) data; Assistive Devices Program data set (ADP). CABG, coronary artery bypass graft surgery; PCI, percutaneous coronary intervention. *OHIP diagnostic codes except written OHIP fee codes. (DOCX) [file pmed.1001599.s006.docx]

**Table S2.** Information about variables derived from administrative data.

| **Components of the primary outcome** | | | | | |
| --- | --- | --- | --- | --- | --- |
| ***Interventions*** | | | | | |
| Inpatient and same day surgery hospitalizations;  code may be recorded in any position | | **The Canadian Classification of Diagnostic, Therapeutic and Surgical Procedure - Until 2001** | | **The Canadian Classification of Health Interventions - Starting in 2002** | |
| ***PCI***  at least one inpatient or same-day surgery record (from CIHI/DAD/SDS*) | | 48.02, 48.03, 48.09 | | 1IJ50, 1IJ57 | |
| ***CABG***  at least one inpatient or same-day surgery record (from CIHI/ DAD/SDS*) | | 48.11 to 48.19 (48.1) | | 1IJ76 | |
| **Diseases** | | | | | |
| **Diseases** | **ICD-9-CM diagnostic codes**  (prior to fiscal year 2002 and) | | **ICD-10-CA code**  (after fiscal year 2002) | | **OHIP codes*** |
| **CV events (primary outcome)** | | | | | |
| ***Hospitalization due to exacerbation of CHF***  most responsible or secondary, including pre-admit comorbidity, discharge diagnosis of CHF in CIHI/DAD | 428.x | | I50 | |  |
| ***AMI***  most responsible diagnosis of AMI in the CIHI/DAD database | 410.x | | I21 | |  |
| ***Stroke***  most responsible diagnosis of stroke in the CIHI/DAD database | **Acute stroke**: 362.3, 430.x, 431.x, 433.x1, 434.x1, 436, 435.x  **Subgroups**:  **Ischemic stroke** (includes acute but ill-defined cerebrovascular): 362.3, 433.x1, 434.x1, 436  **Subarachnoid hemorrhage**: 430.x  **Intracerebral hemorrhage**: 431.x  **Transient cerebral ischemia**: 435.x | | ***Acute stroke***: I60 (excl I60.8);  I61; I63 (excl I63.6); I64; H34.1  G45 (excl G45.4)  **Subgroups**:  **Ischemic stroke** (includes acute but ill-defined cerebrovascular): I63 (excl 163.6), I64, H34.1  **Subarachnoid hemorrhage**: I60 (excl I60.8)  **Intracerebral hemorrhage**: I61  **Transient ischemic attack**: G45 (excl G45.4) | |  |
| **All-causes mortality**  from RPDB, Demographic | the date of the ***death from all-causes*** | |  | |  |
| **Main exposures or risk factors and other potential confounders** | | | | | |
| ***Prior AMI***  Any diagnosis of AMI in CIHI/DAD or OHIP associated with ED visits | 410.x, 412.x | | I21, I22, I25.2 | | 410, 412 |
| ***Prior stroke***  Any diagnosis of stroke in CIHI/DAD or OHIP associated with ED visits | 362.3, 430.x, 431.x, 433.x1, 434.x1, 436, 435.x | | I60 (excl I60.8);  I61; I63 (excl I63.6); I64; H34.1  G45 (excl G45.4) | | 432, 435, 436 |
| ***Prevalent hypertension*** | Diagnostic dates from HIPERTENSION database | |  | |  |
| ***Prevalent CHF*** | Diagnostic dates from Ontario CHF database | |  | |  |
| ***Prevalent diabetes*** | Diagnostic dates from ODD | |  | |  |
| ***Prevalent COPD*** | Diagnostic date from COPD_SENSITIVE2010 | |  | |  |
| ***Prior depression***  hospitalizations where the most responsible diagnosis is major depression from CIHI/DAD, and from OHIP dataset | 296.2, 296.3, 296.5, 300.4, 309.x, 311 | | F20.4, F31.3-F31.5, F32.x, F33.x, F34.1, F41.2, F43.2 | | depression (311) reactive depression (300) |
| ***Prevalent cancer*** | Diagnostic dates from OCRD | |  | |  |
| **Urban/rural status** | ***Urban/rural status*** at time of index diagnostic sleep study, from %getdemo | | | |  |
| **Income**  based on the patient’s postal code and Statistics Canada Postal Code Conversion File | Ontario neighbourhood income are classified into one of five approximately equal-sized groups (quintiles), ranked from poorest (Q1) to wealthiest (Q5) | | | |  |
| **CPAP claims**  from ADP, from Apr 2004 to the last date of follow-up | Types of device prescribed were  CONTINUOUS PAP SYSTEM or "RESMED S8 COMPACT CPAP SYSTEM" or "RESMED S8 ELITE CPAP SYSTEM". | |  | |  |

**List of datasets used**: The Registered Persons Database (RPDB), Canadian Institute for Health Information Discharge Abstract database (CIHI-DAD) and the Same Day Surgery (CIHI-SDS), Ontario Health Insurance Plan Physician Services Claims database (OHIP), NACRS - National Ambulatory Care Reporting System, the Ontario Diabetes Database (ODD), HYPERTENSION, Ontario Congestive Heart Failure database (CHF), Ontario Chronic Obstructive Pulmonary Disease database, Ontario Mental Health Reporting System (OMHRS) stand-alone admissions dataset, Ontario Cancer Registry Data (OCRD), Ontario Registrar General Death (ORGD) data, Assistive Devices Program dataset (ADP).

AMI – acute myocardial infarction, CABG - coronary artery bypass graft surgery; CHF – chronic heart failure; COPD – chronic obstructive pulmonary disease; CPAP – continuous positive airway pressure; PCI - percutaneus coronary intervention
