# Supplementary material for: Obstructive Sleep Apnea and Risk of Cardiovascular Events and All-Cause Mortality: A Decade-Long Historical Cohort Study
Source: PLoS Med. 2014 Feb 4;11(2):e1001599. doi: 10.1371/journal.pmed.1001599 (PMC3913558; doi:10.1371/journal.pmed.1001599)
Supplement: Table S3 — A simple points system based on the beta-coefficients from the final transformed model. Points per predictors, total points and corresponding three- and five-year event-free survival and median survival time are presented. Points per unit of linear predictor: 21.59161. Linear predictor units per point: 0.04631428. (DOCX) [file pmed.1001599.s007.docx]

**Table S3**. A simple points system based on the beta-coefficients from the final transformed model. Points per predictors, total points and corresponding 3- and 5-year event-free survival and median survival time are presented.

Points per unit of linear predictor: 21.59161

Linear predictor units per point: 0.04631428

|  | **Categories** | **Points** |  | **Total Points** | **3-year Survival** |
| --- | --- | --- | --- | --- | --- |
| **Daytime Sleepiness** | N | 0 |  | 188 | 0.05 |
|  | Y | 3 |  | 182 | 0.10 |
| **Number of awakening** in TST, events/TST | 0 | 0 |  | 174 | 0.20 |
|  | 50 | 4 |  | 168 | 0.30 |
|  | 100 | 7 |  | 162 | 0.40 |
|  | 150 | 11 |  | 156 | 0.50 |
|  | 200 | 15 |  | 150 | 0.60 |
|  | 250 | 19 |  | 142 | 0.70 |
|  | 300 | 22 |  | 132 | 0.80 |
| **Sleep time with SaO_2_<90%,** minutes | 0 | 0 |  | 116 | 0.90 |
|  | 50 | 8 |  | 100 | 0.95 |
|  | 100 | 10 |  | 65 | 0.99 |
|  | 150 | 12 |  | **Total Points** | **5-year Survival** |
|  | 200 | 15 |  | 177 | 0.05 |
|  | 250 | 17 |  | 172 | 0.10 |
|  | 300 | 20 |  | 164 | 0.20 |
|  | 350 | 22 |  | 158 | 0.30 |
|  | 400 | 25 |  | 152 | 0.40 |
| **Total sleep time**, hours | 0 | 22 |  | 146 | 0.50 |
|  | 1 | 20 |  | 139 | 0.60 |
|  | 2 | 17 |  | 131 | 0.70 |
|  | 3 | 15 |  | 121 | 0.80 |
|  | 4 | 12 |  | 105 | 0.90 |
|  | 5 | 10 |  | 89 | 0.95 |
|  | 6 | 7 |  | 54 | 0.99 |
|  | 7 | 5 |  | **Total Points** | **Median Survival Time (years)** |
|  | 8 | 2 |  | 188 | 0.5 |
|  | 9 | 0 |  | 177 | 1.0 |
| **Mean heart rate**, bpm | 30 | 0 |  | 164 | 2.0 |
|  | 40 | 4 |  | 156 | 3.0 |
|  | 50 | 8 |  | 150 | 4.0 |
|  | 60 | 12 |  | 146 | 5.0 |
|  | 70 | 16 |  | 142 | 6.0 |
|  | 80 | 19 |  |  |  |
|  | 90 | 23 |  |  |  |
|  | 100 | 27 |  |  |  |
|  | 110 | 31 |  |  |  |
|  | 120 | 35 |  |  |  |
|  | 130 | 39 |  |  |  |
| **Periodic leg movement index** in TST, events/hour | 0 | 0 |  |  |  |
|  | 50 | 4 |  |  |  |
|  | 100 | 7 |  |  |  |
|  | 150 | 11 |  |  |  |
|  | 200 | 15 |  |  |  |
|  | 250 | 18 |  |  |  |
|  | 300 | 22 |  |  |  |
|  | 350 | 26 |  |  |  |
| **Sex** | F | 0 |  |  |  |
|  | M | 10 |  |  |  |
| **Age**, years | 10 | 0 |  |  |  |
|  | 20 | 11 |  |  |  |
|  | 30 | 22 |  |  |  |
|  | 40 | 33 |  |  |  |
|  | 50 | 44 |  |  |  |
|  | 60 | 56 |  |  |  |
|  | 70 | 67 |  |  |  |
|  | 80 | 78 |  |  |  |
|  | 90 | 89 |  |  |  |
|  | 100 | 100 |  |  |  |
| **Smoking Status** | current | 7 |  |  |  |
|  | ex | 1 |  |  |  |
|  | never | 0 |  |  |  |
| **Prior hypertension** | 0 | 0 |  |  |  |
|  | 1 | 6 |  |  |  |
| **Prior stroke** | 0 | 0 |  |  |  |
|  | 1 | 12 |  |  |  |
| **Prior myocardial infarction** | 0 | 0 |  |  |  |
|  | 1 | 8 |  |  |  |
| **Prior chronic heart failure** | 0 | 0 |  |  |  |
|  | 1 | 20 |  |  |  |
| **Prior diabetes** | 0 | 0 |  |  |  |
|  | 1 | 12 |  |  |  |
| **Prior chronic obstructive pulmonary disease** | 0 | 0 |  |  |  |
|  | 1 | 7 |  |  |  |
